# Organoclay flocculation as a pathway to export carbon from the sea surface

**DOI:** 10.1038/s41598-024-79912-z

**Published:** 2024-12-10

**Authors:** Diksha Sharma, Vignesh Gokuladas Menon, Manasi Desai, Danielle Niu, Eleanor Bates, Annie Kandel, Erik R. Zinser, David M. Fields, George A. O’Toole, Mukul Sharma

**Affiliations:** 1 Department of Earth Sciences, Dartmouth College, New Hampshire, USA; 2 Bigelow Laboratory of Ocean Sciences, Maine, USA; 3 Department of Microbiology, University of Tennessee, Tennessee, USA; 4 Geisel School of Medicine at Dartmouth, New Hampshire, USA; 5 Present Address: First-Year Innovation and Research Experience (FIRE), Office of the Senior Vice President and Provost, University of Maryland, College Park, MD USA; 6 Present Address: Department of Oceanography, University of Hawaii at Manoa, Honolulu, HI USA; 7 Laboratoire d’Océanographie et du Climat, CNRS-IRD MNHN- Sorbonne University, Paris, France; 8 Department of Marine Sciences, University of Gothenburg, Göteborg, Sweden

**Keywords:** Clay minerals, TEP, Phytoplankton, Bacteria, Dinoflagellates, Diatoms, Zooplankton, Fecal pellets, Marine carbon cycle, Ocean sciences, Marine biology, Marine chemistry

## Abstract

Marine microorganisms play a critical role in regulating atmospheric CO_2_ concentration via the biological carbon pump. Deposition of continental mineral dust on the sea surface increases carbon sequestration but the interaction between minerals and marine microorganisms is not well understood. We discovered that the interaction of clay minerals with dissolved organic matter and a γ-proteobacterium in seawater increases Transparent Exopolymer Particle (TEP) concentration, leading to organoclay floc formation. To explore this observation further, we conducted a microcosm experiment using surface seawater collected from the Spring 2023 phytoplankton bloom in the Gulf of Maine. Unfiltered (natural community) and filtered (200 μm and 3 μm) seawater was sprayed with clay (20 mg L^− 1^ and 60 mg L^− 1^) and incubated. All clay treatments led to a tenfold increase in TEP concentration. 16S rRNA gene amplicon sequence analyses of seawater and settled organoclay flocs showed the dominance of α-proteobacteria, γ-proteobacteria, and Bacteroidota. The initial seawater phytoplankton community was dominated by dinoflagellates followed by a haptophyte (*Phaeocystis* sp.) and diatoms. Following clay addition, dinoflagellate cell abundance declined sharply while diatom cell abundance increased. By analyzing organoclay flocs for 18S rRNA we confirmed that dinoflagellates were removed in the flocs. The clay amendment removed as much as 50% of phytoplankton organic carbon. We then explored the fate of organoclay flocs at the next trophic level by feeding clay and phytoplankton (*Rhodomonas salina*) to *Calanus finmarchicus*. The copepod ingested *R. salina* and organoclay flocs and egested denser fecal pellets with 1.8- to 3.6- fold higher sinking velocity compared to controls. Fecal pellet density enhancement could facilitate carbon sequestration through zooplankton diel vertical migration. These findings provide insights into how atmospheric dust-derived clay minerals interact with marine microorganisms to enhance the biological carbon pump, facilitating the burial of organic carbon at depths where it is less likely to exchange with the atmosphere.

**Supplementary Information:**

The online version contains supplementary material available at 10.1038/s41598-024-79912-z.

## Introduction

The marine biological pump removes CO_2_ from the atmosphere and stores it in the deep ocean^1^, thereby helping to regulate Earth’s climate by reducing the greenhouse effect. The pump is initiated when phytoplankton, microscopic marine algae, perform photosynthesis using sunlight, water, and carbon dioxide to produce organic carbon compounds, removing 80–146 Pg of atmospheric CO_2_ (1Pg CO_2_ = 1 billion metric tons of CO_2_) annually in the form of dissolved and particulate organic matter^2,3^. The process is highly inefficient, however, as over 90% of the captured carbon is oxidized (‘remineralized’) by marine microbes and the resulting dissolved inorganic carbon (DIC) outgasses as CO_2_ from the sea surface back into the atmosphere^3,4^. The timing of the outgassing varies depending on the particle size. Phytoplankton exudates make up neutrally buoyant dissolved organic matter (DOM), which consists of carbohydrates, proteins, lipids, and other organic molecules. The DIC released during the remineralization of the DOM exchanges with the atmosphere in a matter of days. If the organic matter forms large aggregates or is ingested by zooplankton it may persist in the ocean for a longer time. Zooplankton consume phytoplankton and egest undigested organic/inorganic matter as fecal pellets. Particulate organic matter (POM) consists of zooplankton fecal pellets, phytoplankton aggregates, and aggregates of organic and inorganic detritus. The sequestration time of DIC from remineralization of POM is a function of depth, varying from zero at the sea surface to ~ 400 years at a depth of ~ 1000 m^3^.

Continental mineral dust deposited on the sea surface strengthens the marine biological pump and removes atmospheric CO_2_^5^. Dissolution of mineral dust in seawater provides essential nutrients (phosphate, silica, iron, nitrate) and enhances primary productivity^6,7^. Mineral dust incorporated in organic aggregates also increases their settling velocity via ballasting^8^, potentially enhancing the carbon export from the mixed layer (“export production”), as evidenced by a direct relationship between lithogenic and particulate organic carbon fluxes in the North Atlantic^9^ and the Mediterranean^10^. An increase in the settling velocity of POM would cause remineralization to occur at a greater depth, effectively reducing atmospheric CO_2_^11^. That the deposition of continental mineral dust in the ocean removed CO_2_ is evident over glacial-interglacial timescales. Starting around 40,000 years ago, an increase in the dust flux to the Southern Ocean may have removed as much as 80 Pg of atmospheric CO_2_ suppressing its atmospheric concentration to 180 ppm during the Last Glacial Maximum^12^. Indeed, a strong coupling between dust flux and climate has been observed over the past 800,000 years^13^. However, the exact mechanism of how dust is incorporated in rapidly settling aggregates has not been studied in detail.

We investigated the role of clay minerals in strengthening the marine biological pump. The clay minerals make up > 50% of continental mineral dust^14^ and have surfaces that are charged, rough, and hydrophilic. They could sorb organic molecules dissolved in seawater and attach to phytoplankton exudates such as gel-like adhesive particles known as Transparent Exopolymer Particles (TEP)^15^. Being charged entities, clay minerals react with different types of organic compounds in both terrestrial as well as in marine ecosystems^16–18^. Depending on the structure and chemical composition of organic matter, a variety of interactions can occur that result in the organic matter adhering to the clay mineral surface such as intercalation (penetration of organic molecule into the interlayer space), ligand/ion exchange, cation bridging, grafting reactions (formation of covalent bonds between organics and reactive surface groups), Van der Waals forces and hydrophobic interactions^19^. The interaction of organic molecules with clay results in their being associated with each other from nanometer to millimeter scales with clay minerals shielding the organics from degradation^20–22^.

We surmised that clay minerals sorb DOM, forming organoclay composites that adhere to each other^23^ and to marine gels/TEPs forming aggregates and flocs^24^, which, in turn, grow further as they sink by recruiting bacteria/phytoplankton and by differential settling^25^. We further hypothesized that larger metazoans may ingest organoclay flocs when feeding on phytoplankton. The addition of lithogenic materials including clay may enhance the sinking rates of zooplankton fecal pellets by providing ballast to the fecal pellets^26,27^. This process has the potential to curb the dissolution and degradation of POM during its descent, thereby intensifying the particle sinking flux within the mesopelagic and bathypelagic zones. Thus, the deposition of dust on the sea surface could strengthen the biological pump by transforming DOM to POM and by increasing the depth of remineralization of POM.

We tested the above hypotheses by investigating the interaction among natural clay minerals, bacterioplankton, phytoplankton, and zooplankton. We explored whether clay mineral surfaces primed with DOM would trigger attachment of bacteria/phytoplankton leading to the formation of aggregates and ultimately to rapidly settling organoclay flocs. We next assessed whether zooplankton when presented with organoclay flocs and algae ingest both and egest fecal pellets that settle faster than the controls. Our findings help in understanding how the deposition of continental mineral dust on the sea surface could trigger removal of atmospheric CO_2_.

## Results

### Clay minerals, DOM, and bacteria together contribute to maximal floc formation

To investigate the interplay between clay minerals and marine carbohydrates with marine heterotrophic bacteria, we first identified a mixture of clay minerals (palygorskite and nontronite, Table S1) that effectively sorbs dissolved acidic polysaccharides in synthetic seawater (Fig. S1). These organoclay composites exhibit flocculation and rapid settling through separatory funnels (Fig. S2). In addition, organoclay flocs form in the presence of *Alteromonas* sp. EZ55, which is a marine γ- proteobacterium^28^ specializing in degrading POM associated with phytoplankton bloom^29^. We found that this marine heterotrophic bacterium is over 10 times more abundant in organoclay flocs than in seawater (Fig. S2). This observation was further elucidated in a roller tank experiment, where clay minerals were added to 0.2 μm filtered natural seawater inoculated with *Alteromonas* sp. EZ55 and TEP concentration, floc formation, and bacterial growth were monitored at different time steps (Fig. S3). The data suggest that *Alteromonas* sp. EZ55 facilitates TEP production when exposed to clay and enhances organoclay floc formation, a phenomenon previously unrecognized.

### Impact of clay supplementation on nutrients in a phytoplankton bloom

**Fig. 1:**
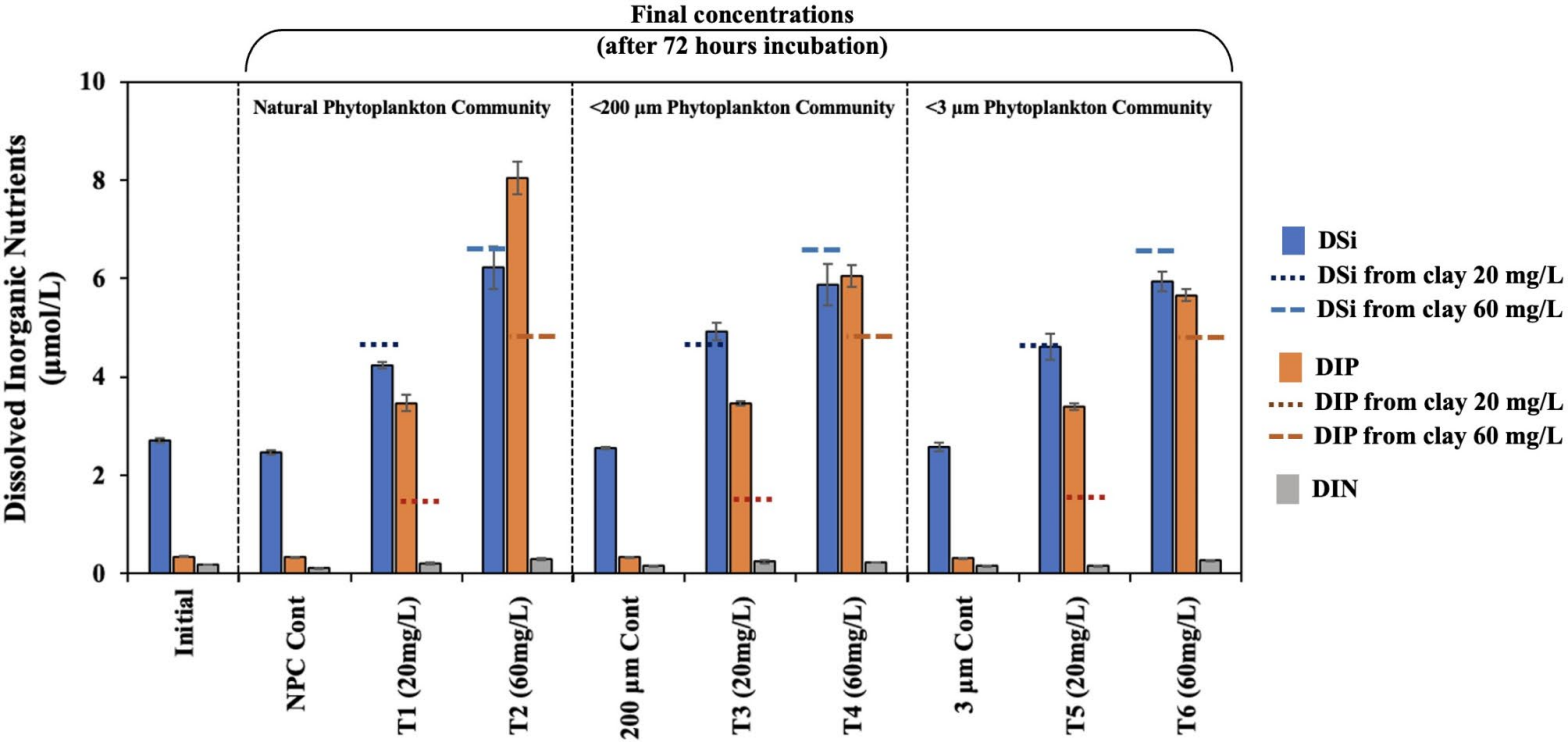
Dissolved inorganic nutrient concentrations (µmol L^− 1^) of initial, controls, and clay-treated seawater. Surface seawater (Initial) was filtered to 200 μm and 3 μm. The unfiltered (Natural Phytoplankton Community, NPC-Cont) and filtered (< 200-PC-Cont and < 3-PC-Cont) water samples were acclimated for 24 h and then treated with two different amounts of clay slurry treatments (T1, T3, T5 = 20 mg clay L^− 1^; T2, T4, T6 = 60 mg clay L^− 1^). Following the clay treatment, the samples (T1 through T6) and controls (NPC-Cont, < 200-PC-Cont and < 3-PC-Cont) were incubated with nutrients measured subsequently. In a separate set of experiments, 0.2 μm filtered seawater amended with 20 mg L^− 1^ and 60 mg L^− 1^ clay was incubated for 72 h. Nutrients leached from clay at the end of this experiment are shown as horizontal dashed lines. There is a clay-dose dependent increase in silica for all clay-treated samples. Leaching of phosphorus associated with clay led to an increase in phosphate in all treated samples. More DIP is released than that expected to leach from clay in 0.2 μm filtered seawater, which may suggest bacterial activity in removing phosphorus from clay and minor amounts of apatite associated with it. Treatment values presented here are the average of samples (*n* = 3). One-way ANOVA (*p* < 0.05) was conducted to investigate the variation in nutrient concentrations influenced by clay compared to the control conditions.

To extend the above observations, we conducted a microcosm experiment aboard *RV Endeavor* in the Gulf of Maine (GoM, 43°33.86’N, 68°29.99’N) during the spring of 2023 phytoplankton bloom. We collected seawater from a depth of ~ 1 m and added clay to unfiltered (Natural Phytoplankton Community, NPC) and filtered (< 200 μm phytoplankton community, < 200-PC and < 3 μm phytoplankton community, < 3-PC) seawater in twenty-seven 1 L polycarbonate bottles that were then incubated for 72 h at ambient seawater temperature in natural sunlight (Fig. S4). The dissolved inorganic nutrient concentrations of unamended seawater were low (dissolved inorganic silicate, DSi = 2.71 ± 0.04 µmol L^− 1^ (instrumental uncertainty given here and subsequently in the paper is 1-σ standard deviation; *n* = 3); dissolved inorganic nitrate plus nitrite, DIN = 0.18 ± 0.01 µmol L^− 1^; dissolved inorganic phosphate, DIP = 0.35 ± 0.01 µmol L^− 1^) (Fig. 1; Table S2). Following incubation, the DSi concentrations remained the same or declined somewhat in all controls. In comparison, DSi concentrations increased in clay-treated unfiltered (Treatment 1, T1 and Treatment 2, T2) and filtered (Treatment 3, T3, Treatment 4, T4, Treatment 5, T5, and Treatment 6, T6) samples (Fig. 1). The DIP increased significantly (*p* < 0.05) in all samples post-incubation when amended with clay.

We conducted a separate experiment to examine the effects of clay leaching into 0.2 μm filtered seawater and found that the introduction of clay resulted in a dose-dependent increase in DSi and DIP levels over a 72-hour period, with no change in DIN concentration (Table S3). This experiment suggests that the observed increase in DSi and DIP concentrations in the clay-amended treatments in the microcosm experiment is likely due to the release of these macronutrients from the clay itself.

### Clay supplementation leads to floc formation with no change in bacterial population

**Fig. 2:**
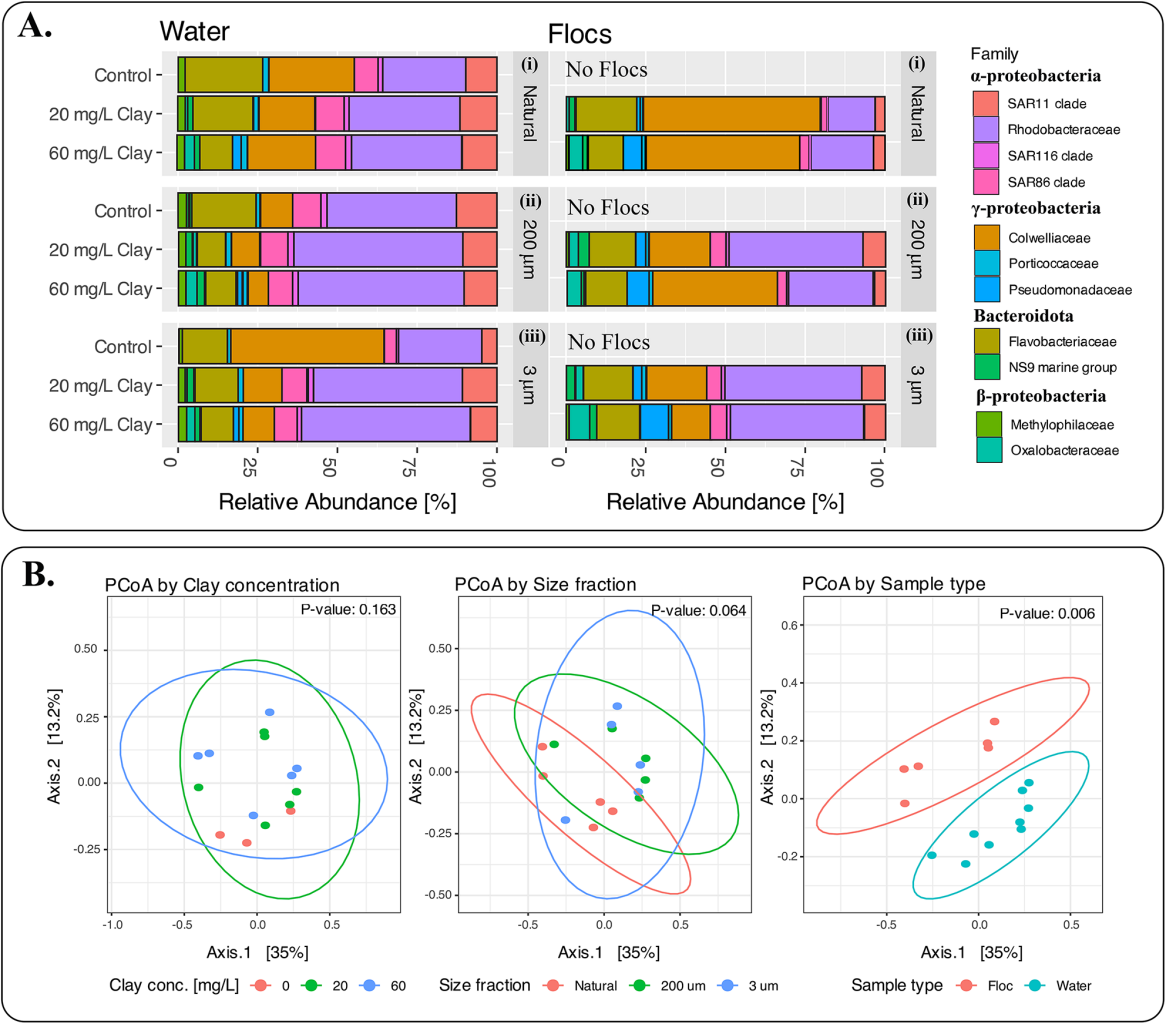
Relative abundance and beta diversity from the 16s rRNA gene amplicon sequencing of bacterial communities in water and floc samples. (A) Relative abundance of bacterial families in the size fractionated microcosm samples (Natural, < 200 μm, and < 3 μm) exposed to varying clay concentrations (0 mg/L, 20 mg/L, and 60 mg/L) show that α-proteobacteria (Rhodobacteraceae), γ-proteobacteria (Colwelliaceae), and bacteroidota (Flavobacteriaceae) dominated both in the water and flocs. (B) Principal Coordinate analysis (PCoA) of beta diversity (Bray-Curtis method) of the bacterial communities based on clay concentrations, size fractions and sample type (floc-associated or suspended/water). Microbial communities showed significant variations only between the flocs and water (*p* < 0.05), but no significant variations with varying clay concentrations or size fractions(*p* > 0.05).

Following incubation, flocs were visible in seawater bottles amended with clay and not in the controls. The initial bacterial abundance in the seawater from the phytoplankton bloom was 2.04 × 10^8^ cells L^− 1^. Following incubation, the bacterial abundances in the controls were 2.5 × 10^8^ cells L^− 1^ (NPC), 1.8 × 10^8^ cells L^− 1^ (< 200-PC), and 1.55 × 10^8^ cells L^− 1^ (< 3-PC). Sequencing the 16S rRNA gene amplicon library of the bacterial community shows that both water and flocs were dominated by bacteria belonging to Rhodobacteraceae, SAR 11, Colwelliaceae, and Flavobacteriaceae clades (Fig. 2A.). The bacterial community in seawater was dominated by heterotrophs belonging to α-proteobacteria (Rohodobacteraceae, SAR11, and SAR86), γ-proteobacteria (Colwelliaceae), and Bacteroidota (Flavobacteriaceae). Flocs obtained from clay-treated samples were similarly dominated by α-proteobacteria (Rhodobacteraceae), γ-proteobacteria (Colwelliaceae), and Bacteroidota (Flavobacteriaceae). While the flocs from the clay-treated microcosms displayed a significant variation (*p* < 0.05) in their microbial community in comparison to the seawater, there was no significant variation (*p* > 0.05) among the microbial community with size fractionation or clay treatments (Fig. 2B).

### The production of Transparent Exopolymer Particles (TEP) is enhanced by clay amendment

**Fig. 3:**
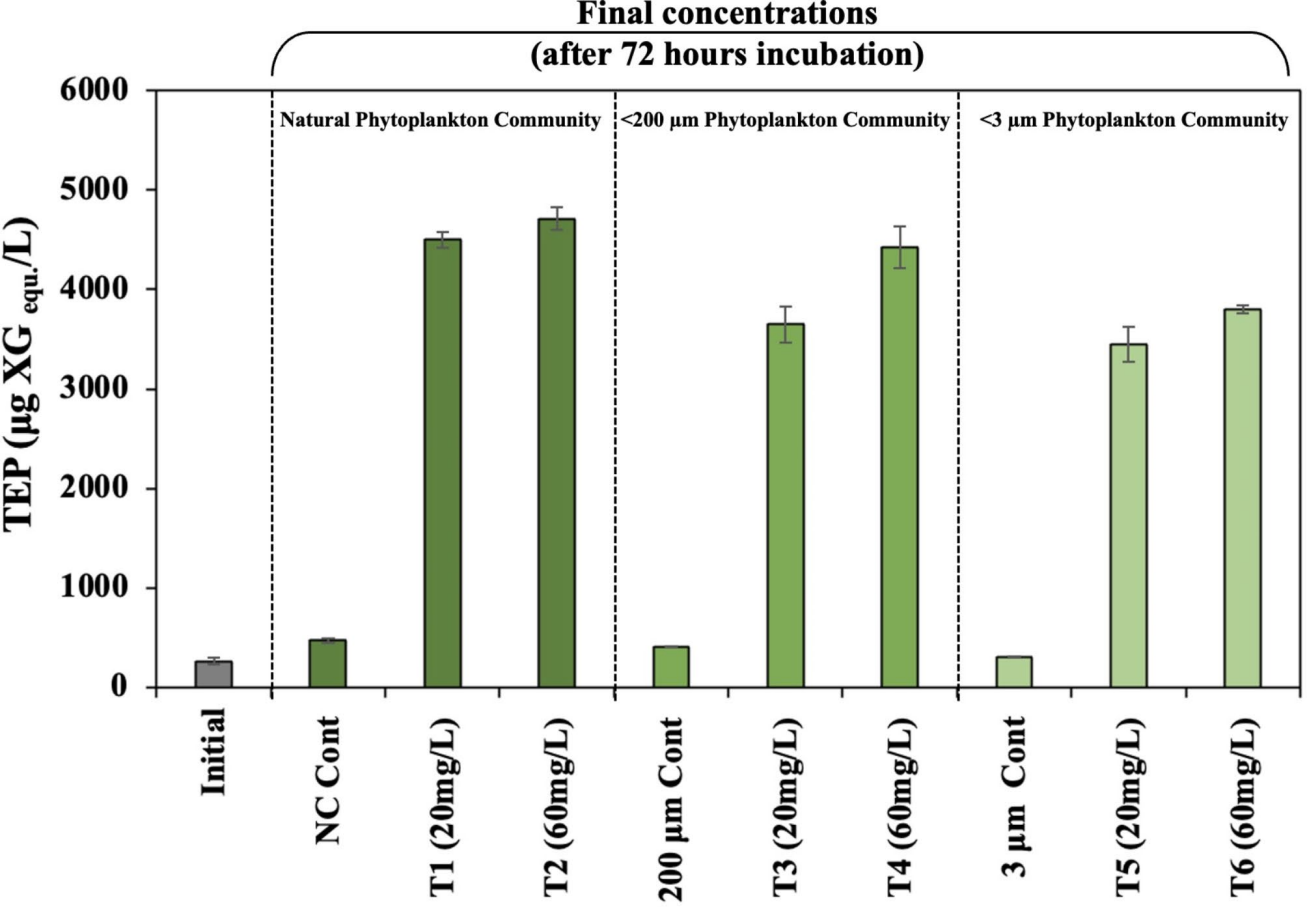
Transparent Exopolymer Particles (TEPs) concentrations of the initial and clay- treated seawater following incubation from the onboard experiment. The TEP concentrations in all three size-separated communities increased over 10-fold over corresponding controls in all clay-treated samples. Refer to Fig. 1 for x-axis labels. Treatment values presented here are the average of samples (*n* = 3). One-way ANOVA (*p* < 0.05) was conducted to investigate the variation in nutrient concentrations influenced by clay compared to the control conditions.

The initial TEP concentration was 0.26 ± 0.03 µg XG eq. L^− 1^ (Fig. 3). In the post-incubated samples in the absence of clay amendment, TEP concentrations increased ≈ 1.5-fold (*p* < 0.05) in NPC controls (0.47 ± 0.02 µg XG eq. L^− 1^) and < 200-PC controls (0.41 ± 0.03 µg XG eq. L^− 1^) but remained constant in the < 3-PC controls (0.30 ± 0.01 µg XG eq. L^− 1^). Clay amendments significantly (*p* < 0.05) enhanced the TEP concentrations in all three communities (NPC, < 200-PC, and < 3-PC) to over 3500 µg XG eq. L^− 1^. There appears to be little or no evidence of clay-dose dependence on the production of TEP. To understand the ambient TEP concentration in the experimental seawater, a separate experiment was conducted in which 0.2 μm filtered seawater was incubated following amendments with 20 mg L^− 1^ and 60 mg L^− 1^ clay. Here we found no increase in TEP concentration , indicating that bacteria are required for TEP production.

### Total chlorophyll a (TChla) is decreased following clay addition

**Fig. 4:**
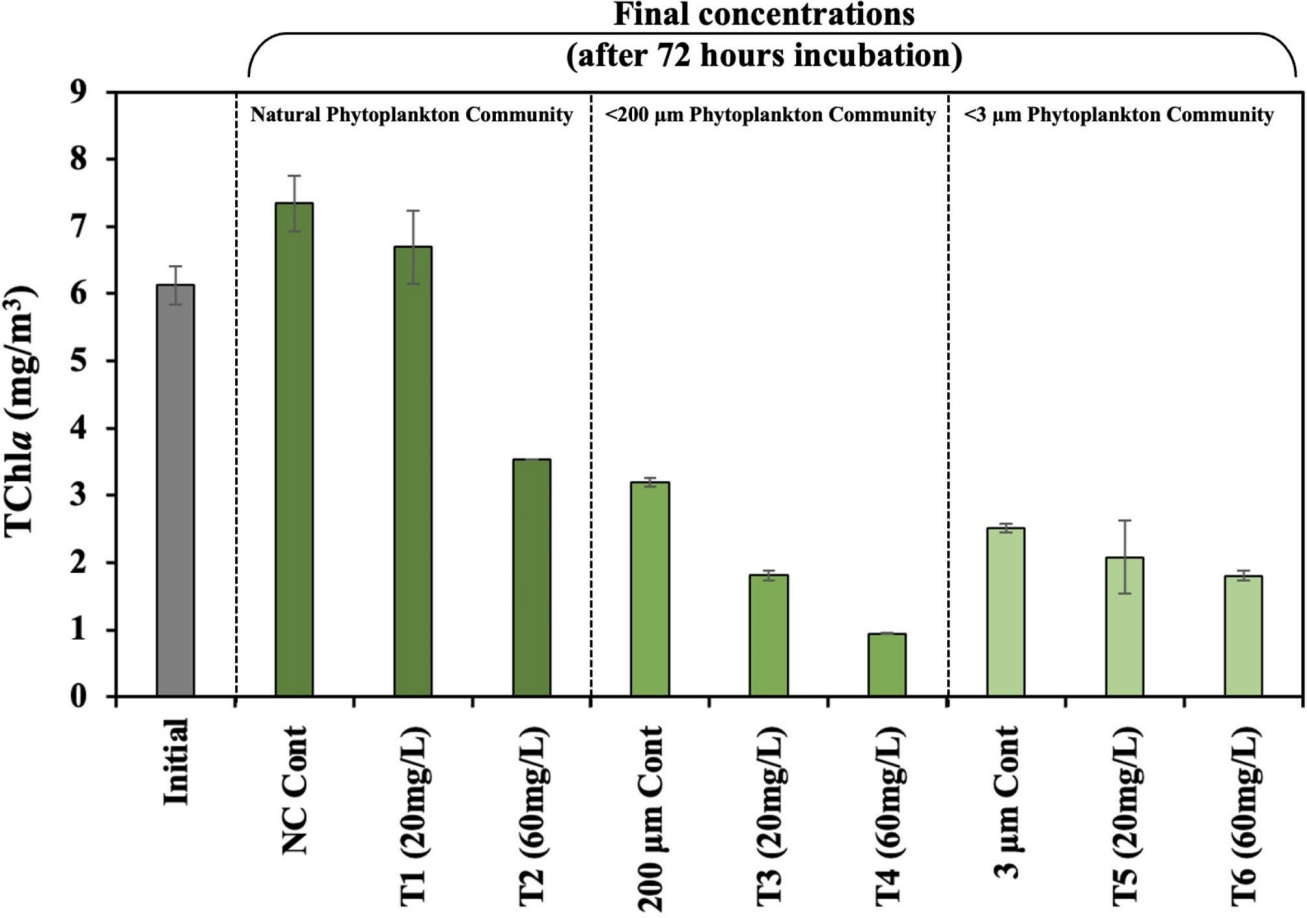
Total Chlorophyll *a* (TChl*a*) concentrations of the initial and clay treated seawater following incubation from the incubation onboard experiment. The TChl*a* concentrations exhibit a decrease in the clay treated samples and a sequential decrease with the decreasing size fraction. Refer to Fig. 1 for x-axis labels. Treatment values presented here are the average of samples (*n* = 3). One-way ANOVA (*p* < 0.05) was conducted to investigate the variation in nutrient concentrations influenced by clay compared to the control conditions.

TChl*a* increased 1.2-fold (*p* < 0.05) in NPC controls (from 6.13 ± 0.3 mg m^− 3^ to 7.34 ± 0.4 mg m^− 3^) after incubation (Fig. 4). Clay addition, however, led to a decrease in TChl*a*. TChl*a* concentration for T1 (20 mg L^− 1^) is lower than that for NPC but within the uncertainty. In comparison, TChl*a* concentration decreased by 2-fold (*p* < 0.05) with T2 (60 mg L^− 1^). In < 200-PC controls, The TChl*a* concentration decreased by a factor of 2 to 3.19 ± 0.3 mg m^− 3^, which we attribute to the filtration of experimental seawater through 200 μm filters. With clay addition, the TChl*a* concentrations reduced significantly (*p* < 0.05) in T3 (20 mg L^− 1^) and T4 (60 mg L^− 1^) treatments compared to their respective control (< 200-PC controls). The TChl*a* concentrations further declined to 2.52 ± 0.3 mg m^− 3^ in < 3-PC controls, due to the filtration of the experimental seawater through 3 μm filters. The TChl*a* concentrations remained unchanged in T5 (20 mg L^− 1^) but showed a reduction in T6 (60 clay L^− 1^) treatments compared to their respective controls (< 3-PC controls).

### Dinoflagellates and Phaeocystis sp. are removed in organoclay flocs while diatoms proliferate

**Fig. 5:**
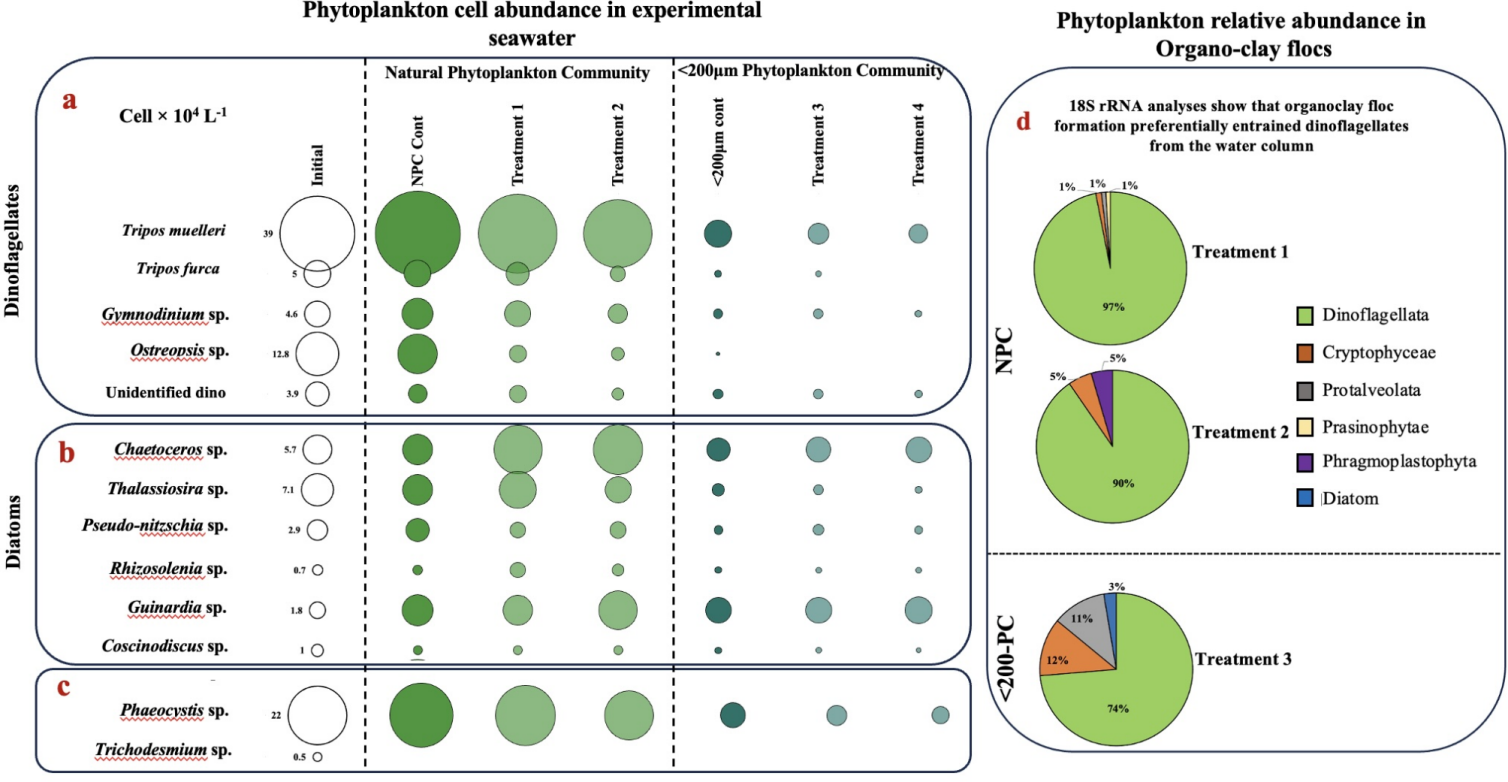
Bubble chart representing the total cell abundance (cell no. × 10^4^ L^− 1^) of the major phytoplankton species analyzed by microscopy in the Natural Plankton Community set of seawater compared to that treated with clay. Treatment values presented here are the average of samples (*n* = 3). Panels a, b, and c give bubble charts of the phytoplankton community as obtained from direct microscopic counting. Panel d represents the relative percentages of phytoplankton present in the organoclay flocs analyzed via 18S rRNA analyses.

 Microscopic analysis of seawater from microcosms showed that the initial phytoplankton community comprised five dinoflagellate, five diatoms, and *Phaeocystis* sp. (a haptophyte), along with cyanobacteria (Fig. 5a, b, c). Dinoflagellates (65.3 ± 0.42 × 10^4^ cells L^− 1^) were the dominant phytoplankton, followed by *Phaeocystis* sp. (22 ± 0.3 × 10^4^ cells L^− 1^), diatoms (19.2 ± 0.85 × 10^4^ cells L^− 1^), and *Trichodesmium* sp. (0.5 ± 0.1 × 10^4^ cells L^− 1^) in the initial community (Table S4). Most abundant among dinoflagellate and diatom species were*Tripos muelleri* (39 ± 2 × 10^4^ cells L^− 1^) and *Thalassiosira* sp. (7.1 ± 0.4 × 10^4^ cells L^− 1^), respectively. Following incubation (NPC controls) the initial phytoplankton cell density increased significantly (*p* < 0.05) from 107 ± 1.1 × 10^4^ cells L^− 1^ to 123.3 ± 2.4 × 10^4^ cells L^− 1^ (Fig. 5, Table S4). The addition of clay caused a significant reduction (*p* < 0.05) in the phytoplankton cell density with respect to the NPC controls. In the < 200-PC control samples, cell density declined to 20 ± 0.9 × 10^4^ cells L^− 1^, which is attributed to the filtration of experimental seawater. Clay addition in the < 200-PC microcosms further reduced the phytoplankton cell density by 20% and 40% in T3 and T4 treatments (*p* < 0.05), respectively, compared to the corresponding controls.

The incubated samples amended with clay showed contrasting trends in the cell abundances of dinoflagellate and diatoms (Fig. 5a, b). The addition of clay resulted in the decline of dinoflagellates. The abundance of *T. muelleri* declined by ≈ 1.5-fold in NPC (T1 and T2) and by ≈ 2-fold in < 200-PC (T3 and T4) (*p* < 0.05). A similar reduction in the cell abundance was also noticed for *Ostreopsis* sp. and *Gymnodinium* sp., with *T. furca* becoming undetectable in all clay-treated samples. The clay addition showed mixed results on the abundances of diatoms in the experimental phytoplankton assemblage. The cell abundance of *Thalassiosira* sp. remained constant in NPC controls in the post-incubated samples. Clay amendment with 20 mg L^− 1^ (T1) resulted in ≈ 1.5 times increased cell abundance (*p* < 0.05). However, further clay addition (T2, 60 mg L^− 1^) resulted in a significant reduction in *Thalassiosira* sp. On the other hand, chain-forming *Chaetoceros* sp. increased ≈ 3-fold (*p* < 0.05) in its cell abundance after clay addition under T1 and T2 treatments compared to its respective control (NPC control). The cell abundance of other large centric diatom *Guinardia* sp. also increased with clay supplementation under T2 compared to its control (NPC controls). Under < 200-PC community, *Thalassiosira*,* Chaetoceros*, and *Guinardia* species showed no significant change in their cell abundances.

The addition of clay also revealed an interesting trend in the cell abundance of the haptophyte *Phaeocystis* sp. (Fig. 5c). A concentration of 20 mg L^− 1^ (T1) showed no significant change, but a higher concentration of 60 mg L^− 1^ (T2) resulted in a 1.7-fold decrease in *Phaeocystis* cell abundance within the NPC group. In the < 200-PC group, clay addition led to a 1.5-fold and 2.2-fold reduction in cell abundance under the T3 (20 mg L^− 1^) and T4 (60 mg L^− 1^) treatments, respectively. 18 S rRNA analyses of the flocs (Fig. 5d) confirmed that organoclay floc formation removes dinoflagellates in the clay-treated groups.

### As much as 50% of phytoplankton organic carbon is removed following clay amendment

 To evaluate phytoplankton organic carbon removed from experimental seawater, phytoplankton cell volumes were determined for seven dominant phytoplankton species (including three dinoflagellates, three diatoms, and *Phaeocystis* sp.) present in both the initial and post-incubation samples. Using previously established carbon to volume relationship^30,31^, the carbon content for these dominant species was calculated. Since these seven species contributed the majority to the total phytoplankton community, their cumulative carbon content was measured as an indicator of the phytoplankton organic carbon pool. Initially, the total carbon content was 3.02 ± 0.18 mg C L^− 1^, which increased to 3.76 ± 0.13 mg C L^− 1^ in the post-incubation NPC control. However, following clay addition, a 15% (*p* < 0.05) reduction in carbon content was observed under the T1 (20 mg L^− 1^) treatment, and a 42% (*p* < 0.05) reduction under T2 (60 mg L^− 1^). In the < 200-PC controls, the carbon content was lower (0.37 ± 0.03 mg C L^− 1^) and further decreased by 41% (*p* < 0.05) and 52% (*p* < 0.05) in the T3 (20 mg L^− 1^) and T4 (60 mg L^− 1^) treatments, respectively, post-incubation.

### Copepods (*Calanus finmarchicus*) egest fecal pellets whose density increases with increasing dosage of clay

**Fig. 6:**
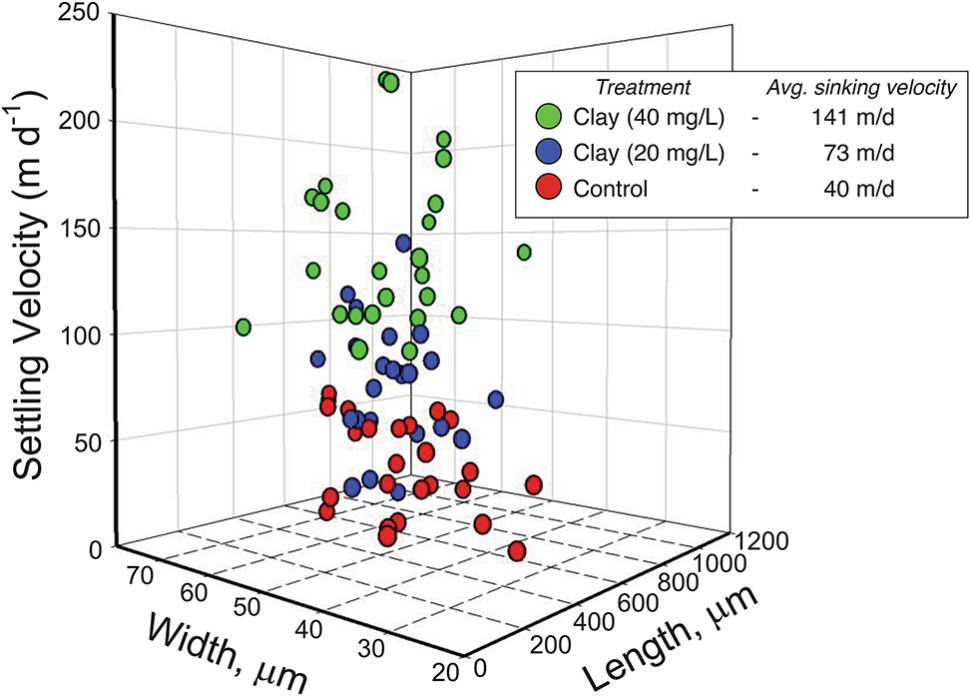
This graph describes the sinking velocity of fecal pellets produced by *Calanus finmarchicus* following feeding on a diet comprising algae *Rhodomonas salina* supplemented with varying concentrations of clay. The legend denotes three distinct treatments: the Control group (red dots) received algae exclusively, while Treatment 1 (blue dots) involved algae supplemented with 20 mgL^− 1^ clay, and Treatment 2 (green dots) incorporated algae supplemented with 40 mgL^− 1^ clay. Treatment values presented here are the average of samples (*n* = 24–25). One-way ANOVA (*p* < 0.05) was conducted to investigate the variation in nutrient concentrations influenced by clay compared to the control conditions.

To investigate whether phytoplankton with clay is ingested by zooplankton (*Calanus finmarchicus)*, about 75 copepod females were incubated in microcosms with two different concentrations of clay (20 and 40 mg L^− 1^) along with identical concentrations of *Rhodomonas salina*, which does not make mineral frustules. The fecal pellet production rate experiment was conducted initially to assess potential differences between the control and clay-treated groups. We have not observed any significant changes between these treatments (Table S5). After this experiment, we separated fecal pellets from the microcosms and determined their sinking rates individually using a high-speed camera. The average settling velocity of *C. finmarchicus* fecal pellets in control, characterized by an exclusive algal diet, was recorded at 40 ± 18 m day⁻¹ (Fig. 6). Upon the introduction of clay into the algal diet, a noteworthy enhancement in the sinking velocity of fecal pellets was observed. Specifically, fecal pellets containing 20 mg L⁻¹ and 40 mg L⁻¹ of clay exhibited sinking velocities of 73 ± 28 m day⁻¹ (*p* < 0.05) and 141 ± 37 m day⁻¹ (*p* < 0.05), respectively. The sinking velocity is independent of the dimensions of the fecal pellets (Fig. 6) and indicates the presence of clay within the fecal pellets. It follows that *C. finmarchicus* ingests organoclay flocs and egests fecal pellets that are denser due to the presence of undigested clay.

## Discussion

Diatoms have been identified as the primary taxonomic group in GoM during the spring bloom, followed typically by a succession of dinoflagellates, coccolithophores, and picophytoplankton^32^. The succession is determined by the nutrient concentrations that vary spatially and temporally in the ambient seawater. Seawater DSi concentration declines as a result of diatom growth, which becomes limited when the concentration reaches < 2 µmol L^− 1^^33^. The average DSi concentration at the time of collection of our experimental seawater was ≈ 2 µmol L^− 1^. Cumulatively, all diatoms in the experimental seawater constituted ≈ 20% of the total cell numbers.

In comparison to diatoms, dinoflagellates have low half-saturation constants for phosphate and nitrate^34–36^ and become dominant as phosphate and nitrate concentrations decline following the demise of a diatom bloom. The Spring 2023 bloom in the GoM was unusual as a novel bloom of large mixotrophic dinoflagellate *T. muelleri* was observed^37^. In our experiment, *T. muelleri* was also dominant. All dinoflagellate species combined exceeded 60% of the cell abundance (Fig. 5). The ambient TChl*a* concentration was quite high (≈ 6 mg m^− 3^), implying that the dinoflagellate bloom had likely reached the exponential growth phase (Fig. 4).

Another notable observation in the phytoplankton community is the presence of the haptophyte *Phaeocystis* sp., a cosmopolitan, bloom-forming mixotroph that contributes significantly to global carbon and sulfur cycles^38^. This species was the second most dominant after the dinoflagellate *T*. *muelleri* and constituted ≈ 20% of the total cell counts. *Phaeocyctis* sp. exists as individual cells as well as in colonies^39^. However, only individual cells were observed microscopically in our experimental seawater. Studies have shown that low silica conditions limiting diatom growth free up nitrate facilitating the growth of haptophytes^39–42^. The co-existence of *T. muelleri* and *Phaeocystis*, both requiring nitrate, is intriguing and may suggest that they may have different nutrient kinetics because of different cell sizes (*T. muelleri* = 200 μm and *Phaeocystis* = 3–8 μm).

Following the clay enrichment, there was a significant change in the phytoplankton community structure, resulting in an increased proportion of diatoms over dinoflagellates and *Phaeocystis* sp. (Fig. 5). Previous nutrient enrichment studies have observed that diatoms become the predominant phytoplankton following the addition of nutrients^43,44^. This is attributed to diatoms’ superior capacity to rapidly utilize new nutrient inputs^45^ and their ability to avoid grazing pressure^44^. We found that *Guinardia* sp. and *Chaetoceros* sp., proliferated following clay amendment. These species are known for their high growth rates under increased nutrient conditions^46^ and proliferate by aggressively ingesting nutrients^47^, including the new nutrients as they become available^45^. The growth rates of diatoms must be supported by silica^48^ which was provided by the clay (Fig. 1). As diatoms grow, they compete with *Phaeocyctis* for nitrate^49^. The observed reduction in *Phaeocystis* cell abundance following clay amendments (Fig. 5c) could be due to the competition by diatom or due to the removal of cells via organoclay flocs. Organoclay floc formation removed several phytoplankton species and reduced the total cell number (Table S4). 18S rRNA analyses of flocs extend microscopic observations revealing that along with microplankton (dinoflagellates, diatoms, Phragmoplastophytes), the flocs removed nanoplankton belonging to Cryptophyceae, Protalveolata, and Prasinophyceae (Fig. 5d). Interestingly, *Phaeocystis* sp. is not present in the samples analyzed for 18S rRNA. This could be due to the absence of *Phaeocystis* colonies, which are mucilaginous and tend to sink^39^.

Within the < 3-PC community, TChl*a* levels decreased somewhat with the addition of 20 mg L^− 1^ clay, whereas a significant decrease was observed with 60 mg L^− 1^ clay (Fig. 5). Although we do not have flow cytometry data to directly assess the response of picophytoplankton to clay addition, we can infer some trends from the TChl*a* data in the < 3-PC community. The dominant photooxygenic picoplankton in GoM is *Synechococcus*^50^. The TChl*a* data suggest that at higher (60 mg L^− 1^) clay concentration, *Synechococcus* may have been removed by the organo-clay flocs, consistent with the observations by Deng et al.^51^.

In the Gulf of Maine waters, the dinoflagellate species exceeded 60% of the cell abundance (Fig. 5), indicating that the diatom bloom likely had collapsed a few days prior to our sampling. In the event of a bloom collapse, organic molecules released by the lysing of cells often combine with dying/dead phytoplankton to form aggregates^24^. This particle aggregation process is facilitated by TEP, which are composed of acidic polysaccharides^15,24^. The formation of TEP is a complex process, impacted by a variety of biotic and abiotic processes^4^. Indeed, our in vitro studies suggested that heterotrophic bacteria plus clay contributed to the maximal TEP formation (Fig. S3), a finding that was corroborated by GoM microcosm experiments (Figs. 2 and 3).

The measured TEP concentrations in the initial and controls of our incubation experiment were within the range reported in other studies from the North Atlantic Ocean^52^. Surprisingly, however, the TEP concentrations in clay-treated samples increased by a factor of about ten in all three phytoplankton communities (NPC, < 200-PC, and < 3-PC) (Fig. 3). The TEP were measured after the formation of organoclay flocs that were recovered at the bottom of microcosm bottles. It is therefore likely that the TEP concentrations were much higher following the clay addition. Since the TEP concentration increased in all size fractions, we think that the presence of clay induced phytoplankton and bacterioplankton to exude TEP precursors. The dominant bacterioplankton in < 3-PC water and organoclay flocs are heterotrophs belonging to α-proteobacteria, γ-proteobacteria, and Bacteroidota (Fig. 2). It follows that heterotrophic bacteria likely facilitated TEP production when exposed to clay minerals. This conclusion is consistent with our observations of *Alteromonas* EZ55, a γ-proteobacterium that mediates TEP production in the presence of DOM and clay (Figs. S2, S3).

Marine heterotrophic bacteria have long been recognized as fully or partially covered with acidic polysaccharides that promote bacterial adhesion to surfaces^53^. Heterotrophic bacterial communities are believed to colonize and use TEP^54^, and any surface immersed in seawater is quickly covered by a proto-biofilm made of TEP and bacteria^55,56^. Surface attachment creates a biofilm giving an evolutionary advantage to bacteria in an otherwise highly diluted and variable ocean environment^57^. As clay minerals sorb DOM (Figs. S1, S2, S3), the observed enhancement in TEP concentration (Fig. 3) may thus be related to the propensity of phytoplankton-associated marine heterotrophic bacteria to attach to a surface. Our experiments show that an increase in TEPs results in the formation and flocculation of organoclay aggregates, which entangle ambient phytoplankton. Thus, the net effect of the interaction of clay minerals with DOM, heterotrophic bacteria, and phytoplankton is the removal of the phytoplankton by organoclay flocs. This has been demonstrated by the cell volume-based carbon content (mg C L^− 1^) calculated for the phytoplankton cell numbers (Table S2). NPC treatments with 20 and 60 mg L^− 1^ clay reduced microcosm carbon content by 15% and 42%, respectively. This reduction further increased (up to 52%) under < 200-PC treatments following clay (60 mg L^− 1^) addition. It is important to note that this calculated carbon content is derived from the cell volumes of the seven phytoplankton species that dominated the community and represents a lower bound on carbon reduction in the experiment. While these findings support the hypothesis that phytoplankton are removed by organoclay flocs, field studies would be needed to fully understand the magnitude of carbon export following clay addition.

The clay concentrations used in the present study fall within the range of deposition from Saharan dust observed in the North Atlantic Ocean ranging from 5.6 to 56 mg m^− 2^ day^− 1^^58–61^. We also note that individual dust storms can deposit significantly larger amounts of dust^62^. On average, clay minerals are < 1 μm in size, and uniform deposition of just 1 mg of clay per m^2^ to the sea surface would result in the sea surface microlayer (~ 1000 μm thick) having a density of ~ 7000 clay grains per µL. In comparison, 1 µL of seawater in a bloom contains about 500–3000 heterotrophs, along with 10,000 viruses, and less than 100 of each of picocyanobacteria, protists, and algae^63,64^. Thus, strong interaction between clay minerals in dust and microbes is expected. Given that TEP strongly facilitate aggregation, flocculation, and marine snow formation, their production in the presence of clay minerals provides a mechanism whereby the deposition of continental mineral dust on the sea surface aids in removing atmospheric carbon. This removal occurs through photosynthesis and through ballasting the fixed carbon to the deeper ocean.

It is imperative to comprehend the repercussions of organoclay floc aggregation on the next trophic level within the epipelagic and mesopelagic zones, particularly considering its impact on carbon export efficiency. Due to the susceptibility of settling POM to microbial degradation in the mesopelagic, a persistent prevailing belief is that rapid-sinking entities, such as zooplankton fecal pellets, serve as the principal drivers of the downward flux in the ocean^65^. Changes in the diets of zooplankton can influence the sinking rates of the resulting fecal pellets^66^. We found that copepod *C. finmarchicus* ingested organoclay flocs and egested clay-embedded fecal pellets with settling velocities that were 1.8- to 3.6- times higher than controls. Such increases in fecal pellet settling velocity combined with zooplankton diel vertical migration could facilitate carbon sequestration. During these migrations, zooplankton transfer organic carbon into deeper ocean sections. Many mesozooplankton species exhibit diel vertical migration, ascending to surface waters at night to feed and descending during daylight hours to evade visual predators^67^. This behavior often takes them below the euphotic zone, where they release organic matter and CO_2_, actively contributing to export flux^68,69^.

**Fig. 7:**
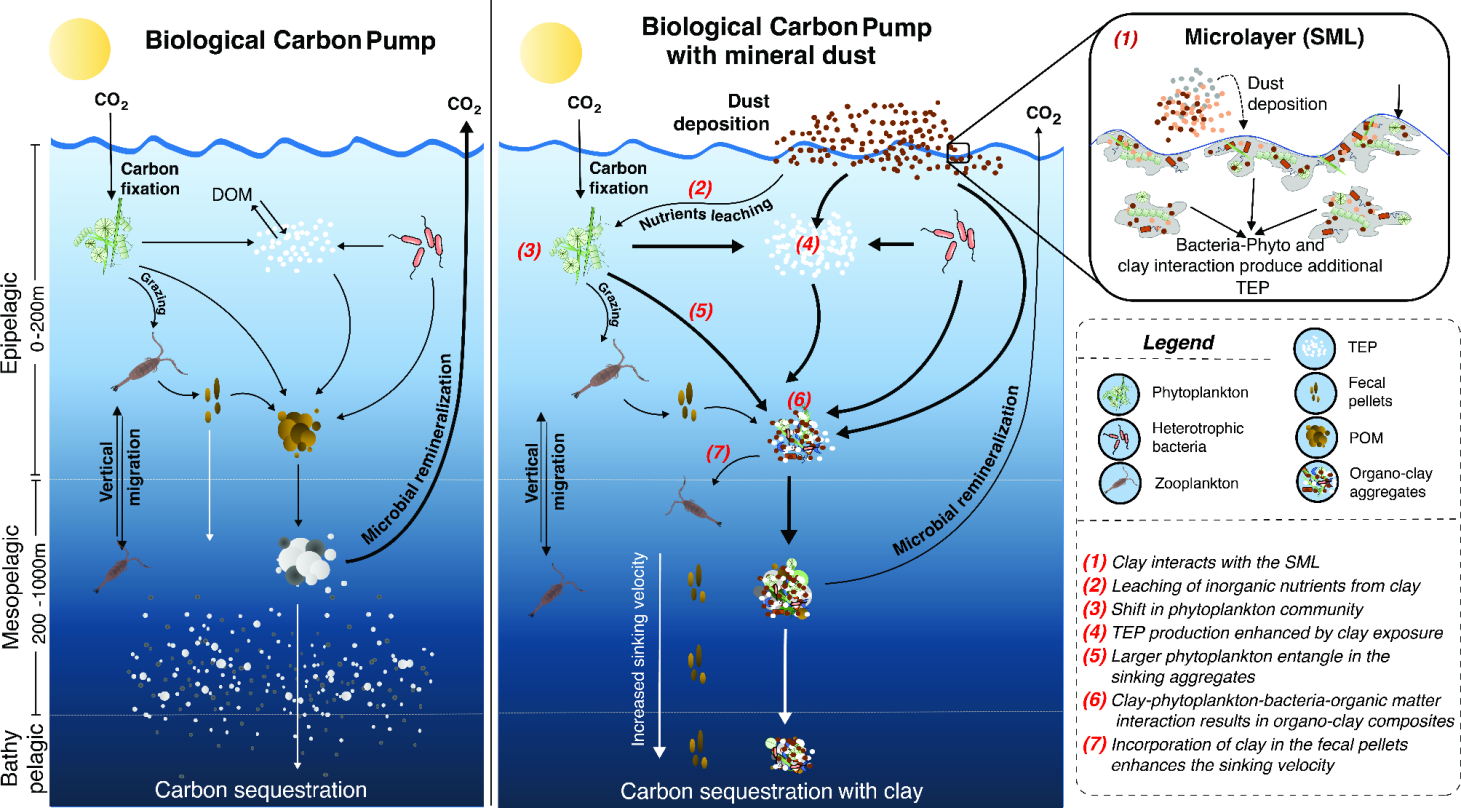
Scheme comparing the Biological Carbon Pump (BCP, left box) with the BCP augmented by deposition of continental mineral dust at the sea surface (right box). The thickness of the arrows is indicative of the strength of the respective pathways. The BCP exports photosynthetically produced organic carbon in the form of sinking particulate organic matter (POM). The POM aggregation is facilitated by acidic polysaccharides exuded from phytoplankton and heterotrophic bacteria that form transparent exopolymer particles (TEPs)/microgels. The BCP is inefficient in removing atmospheric carbon permanently to the ocean sediment as the bulk inventory of POM is remineralized by marine biota during transport through the epipelagic and mesopelagic. Our experiments suggest that deposition of continental mineral dust associated clay minerals at the sea surface recruits the heterotrophic bacteria to produce more TEP and trigger a pathway that strengthens the BCP by (a) converting buoyant dissolved organic matter into settling organoclay flocs, (b) forming and sinking of phytoplankton aggregates in bloom, and (c) mineral ballasting, thereby increasing the mean depth of respiration of POM. Moreover, ingestion of organoclay flocs by microzooplankton repackages clay with carbon resulting in fecal pellets with enhanced settling rates. We posit that high fecal pellet sinking rates combined with diel migration of microzooplankton further boost the sequestration of atmospheric carbon. The above processes can occur in combination with the changes in productivity and organismal community structure resulting from the deposition of mineral dust.

Based on our findings we posit the following explanation for the continental mineral dust enhancing carbon sequestration (Fig. 7): When the dust is deposited on the sea surface over a bloom, many interactions among clay-DOM-heterotrophic bacteria and plankton can be expected. Not only does the dust provide nutrients driving up productivity, but the clay minerals in the dust also provide surfaces that sorb organic molecules dissolved in seawater. Certain bacteria, particularly heterotrophs, can detect and attach to solid surfaces^56^ including those of clay minerals initiating aggregation and organoclay floc formation. Zooplankton feeding on organoclay flocs egest rapidly settling fecal pellets potentially releasing them below the euphotic zone during their daily vertical migration.

The above observations suggest a new approach to remove atmospheric CO_2_ in the ocean by utilizing clay minerals. By spraying a slurry of clay minerals on the sea surface the remineralization depth of photosynthetically fixed carbon can potentially be increased to effectively remove atmospheric CO_2_. Experiments in oceanographically relevant settings will be needed to evaluate the efficiency of carbon export. These settings, for example, are along the continental margins with high primary production: California Current System (primary production off coastal Southern California = 83–450 g C m^− 2^ yr^− 1^^70^), Peru Upwelling System (primary production = 100–400 g C m^− 2^ yr^− 1^^71^), Cariaco basin (primary production = 500 g C m^− 2^ yr^− 1^^71^), and Benguela Upwelling System (total primary production = 1000 g C m^− 2^ yr^− 1^^73–75^). These oceanographic regions hold promise for evaluating the effectiveness of carbon removal through clay seeding and validating this approach.

## Materials and methods

Synthetic and natural seawater samples were sprayed with two different concentrations of natural clay consisting of palygorskite and nontronite with minor apatite to evaluate nutrient and chlorophyll variation, bacterioplankton and phytoplankton abundance, and formation of organoclay aggregates and flocs. Details of clay characterization, seawater collection, filtrations, and incubation experiments are given below. Briefly, we first identified a mixture of clay minerals to be used by changes in their electrophoretic mobility and DOM scavenging capacity. We then investigated floc formation when a heterotrophic bacterium *Alteromonas* sp. EZ55 interacts with clay minerals in synthetic seawater. Further, roller tank experiments were conducted using 0.2 μm filtered water from the Gulf of Maine bloom inoculated with *Alteromonas* sp. EZ55 and clay to confirm that a heterotrophic bacteria can facilitate TEP production and expedite floc formation in the presence of clay minerals (Fig. S3). To investigate floc formation in a phytoplankton bloom, we sampled surface seawater from the Gulf of Maine during the Spring 2023 bloom. Water was filtered to assess the interaction of clay minerals with different communities. Sample bottles containing filtered and unfiltered water were treated with clay slurry and incubated on the deck of the *RV Endeavor* in a water bath for 72 h. The resulting samples were analyzed on the ship for nutrients and TEP levels. Additional analyses, including microscopy, 16 S rRNA, and 18 S rRNA community analyses for bacteria and phytoplankton identification respectively, were performed in onshore laboratory. To deepen our comprehension of how organoclay flocs transfer carbon to the next trophic level, a set of microcosms experiments investigated whether *Calanus finmarchicus* ingest organoclay flocs mixed with an alga (*Rhodomonas salina*). The copepod was fed two different concentrations of clay (20 and 40 mg L^− 1^) along with algae and the sinking rates of fecal pellets were measured.

### Characterization of natural clay

Natural clay, a by-product of mining in Florida, contains a mixture of clay minerals (palygorskite, nontronite), phosphates (apatite, vivianite), quartz, and dolomite. Palygorskite is a fibrous clay mineral that is found associated with Saharan dust^76,77^. Nontronite is an iron-rich clay belonging to smectite group of minerals that has also been observed in Saharan dust originating from Bodéle depression^76,78^. Natural clay was prepared for quantitative X-ray diffraction (XRD) analysis following the procedure by Eberl^79^. Briefly, each sample was ground with corundum (American Elements, 99.9%) and ethanol in a McCrone microionising mill for 5 min. After drying, the sample was shaken in a glass scintillation vial with three 10 mm Teflon balls. A high vapor pressure organic liquid (Vertrel, Fisher Scientific) was then added to the sample, and the contents were shaken for another 10 min with the Teflon balls. If clumping occurred, excess Vertrel was allowed to evaporate in a hood and the sample was shaken again for 10 min with the Teflon balls. This procedure homogenizes the sample and minimizes the potential for a preferred mineral orientation^80^. The sample was backpacked into an XRD holder and scanned with CuKα radiation (40 mA, 40 kV) using a Bruker-AXS D8 Advanced X-ray diffractometer equipped with a Ge crystal monochrometer for XRD analysis. We scanned samples with a step size of 0.02° 2θ from 5–65° and a counting time of 2 s per step and quantified the mineralogical content using the RockJock computer program^79^. RockJock quantitatively determines the mineralogical composition (5% uncertainty) of a sample by comparing the integrated intensities of minerals relative to the corundum standard.

### Preparation of clay for incubation experiments

The clay, initially prepared as a slurry in deionized water, underwent homogenization using an ultrasonicator and was then suspended in a 1 L column of deionized water overnight. Only the top third of the water column with suspended clay was then carefully decanted. This procedure removed the bulk of quartz, dolomite, and apatite. The resulting clay contained palygorskite and notronite in approximately 80:20 ratio. The clay was rinsed in de-ionized water, freeze-dried, and then tested for bacterial contamination on an agar plate. It was found to be sterile. Pre-weighed amounts of clay were then suspended in de-ionized water and used for incubation experiments.

### Clay-acid polysaccharide interaction investigated using ζ potential

To evaluate the extent to which palygorskite and nontronite sorb acidic polysaccharides separately and together, we used palygorskite PFl-1 Mg_5_Si_8_O_20_(OH)_2_(OH_2_)_4_·4H_2_O^81^ and nontronite NAu-1 (Na_1.05_) [Si_6.98_ Al0.95Fe_0.07_][Al_0.36_ Fe_3.61_ Mg_0.04_]O_20_(OH)_4_^82^ obtained from the Source Clays Repository of the Clay Minerals Society. Acid polysaccharide solutions were prepared by adding ~ 40 mg commercial xanthan gum or alginic acid to 250 mL synthetic seawater or de-ionized water. About 2.5 g total clay (palygorskite, nontronite, or an 80:20 mixture) was added to each solution, shaken for 15 min, and left to settle overnight. Samples were then rinsed, freeze-dried, and aliquots were taken for Total Organic Carbon (TOC) measurements. To measure the ζ potential sample aliquots were re-dispersed in 0.01 M NaCl. The ζ potential was measured using a Zetasizer Nanoseries (Malvern Instruments) (Fig. S1).

### Separatory Funnel experiments

Autoclaved separatory funnels (1 L) were used to evaluate the interaction of clay with dissolved organic matter (acid polysaccharide—a mixture of alginic acid and xanthan gum) and a common copiotroph (*Alteromonas* sp. EZ55^28^) in synthetic seawater, which was filtered to 0.2 μm (Fig. S2A). The bacterium was grown separately and then inoculated in separatory funnels. Clay slurry was subsequently sprayed. Sediment was collected after 24 h of shaking, freeze dried, and analyzed for TOC and TON. To determine the total bacterial abundance in the seawater and aggregates, samples were plated as serial dilutions on Marine agar 2216 (BD Difco 2216, Bacton Dickinson, NJ), and incubated at 28 ˚C for 48–72 h until individual colonies were distinguishable to count. The results are reported as colony forming units (CFU)/mL^83^. The serial dilution of samples was conducted in seawater complete liquid medium. Seawater complete medium^84,85^ is prepared as follows (per L): 1000 ml seawater base medium supplemented with Bacto tryptone (5 g), yeast extract (1 g), glycerol (3 mL), 1 M MOPS (5 mL) adjusted to a pH of 7.2. Seawater base medium contains (final concentration): NaCl (242.2 mM), MgCl_2_⋅6H_2_O (1.97 mM), CaCl_2_⋅2H_2_O (0.68 mM), and KCl (6.71 mM).

### Roller tank experiment

Sterile filtered (0.2 μm) seawater collected from the GoM (*RV Endeavor*) was used to understand the interaction between a heterotrophic bacterium, *Alteromonas* sp. EZ55, and clay minerals in a roller tank experiment. *Alteromonas* sp. EZ55 was cultured overnight in Marine Broth 2216 at 28 °C and 200 rpm. An adequate amount of the culture was then aliquoted, washed into sterile seawater, and introduced into the roller tanks to a final concentration of 1 × 10^8^ cells L^− 1^. Seven roller tanks were set up, each at a capacity of 1.15 L (14 cm inner diameter and 7.47 cm length) and rolled at 3 rpm. The treatments were 2 × Clay control (20 mg L^− 1^), 2 × Bacterial control (10^8^ cells L^− 1^), 2 × Bacteria + Clay treatment, and 1 × Seawater control. The tanks were incubated for a total of 24 h and were photographed every hour to monitor flocculation (Fig S3). After the incubation period, the tank was set on its side and the flocs formed were left to settle. The supernatant water in each tank was analyzed for TEP concentrations and bacterial counts. As above, the total bacteria in the seawater and aggregates were determined by serial dilution plating and are reported as CFU/mL (Fig. S3A).

### Sampling Spring 2023 phytoplankton bloom in the Gulf of Maine and incubation

Between April 1–10, 2023, an onboard incubation experiment was conducted on *RV Endeavor* using surface seawater sampled from a depth of ~ 1 m. A towed surface pump system (“tow-fish”)^86^ was used to collect seawater. The tow-fish system consists of an air-actuated PTFE diaphragm pump (Wilden pump & Engineering LLC) and PFA Teflon™-lined tubing connected to a bathythermograph. To ensure its cleanliness, the entire system was cleaned with 10% HCl, followed by rinsing with de-ionized water before deployment. The tow-fish was deployed from the port side of the vessel using a davit and towed at speeds of 7–8 knots, allowing for the collection of uncontaminated surface water with phytoplankton bloom. The ambient surface temperature and salinity were recorded with the help of sensors attached to the CTD (Seabird, USA), which were 10 °C and 30.5, respectively.

For the microcosm experiment, surface seawater was collected and filtered into three size fractions (bulk, < 200 μm, and < 3 μm) in acid-cleaned polycarbonate bottles (Nalgene; 1 L). The first set comprised natural phytoplankton community (NPC) seawater, while the second and third sets were filtered through 200 μm and 3 μm nylon meshes, respectively. A total of 27 bottles were used in this experiment, with nine each for the NPC seawater, < 200 μm community (< 200-PC), and < 3 μm community (< 3-PC). All the bottles were kept in the water bath on the ship deck for the phytoplankton acclimatization for 24 h under a natural light and dark cycle. After 24 h of acclimatization, clay slurry was sprayed uniformly in the head-space of each bottle and allowed to settle. For a given filtered water, clay slurry was spread to achieve two different concentrations of 20 mg L^− 1^ and 60 mg L^− 1^. All bottles were sealed and placed in a water bath on the deck for incubation. The temperature of the water bath was maintained by passing a continuous stream of ambient seawater. The intensity of light was measured hourly using a PAR meter (MQ-200X; Apogee instruments, USA) with an average of 53 µmol Einsteins m^− 2^ day^− 1^ during the incubation period. The bottles were incubated for 72 h and subsequently sampled for further analysis of the final parameters after incubation (Fig S4).

Acid-cleaned centrifuge tubes were used to collect the initial samples for dissolved inorganic nutrients (Silicate, phosphate, nitrate + nitrite), which were then analyzed on board. Initial samples were also collected to identify phytoplankton and determine their cell counts. Separate aliquots of samples were taken to determine TChl*a*, transparent exopolymer particles (TEPs), and total bacterial counts (TBC). These samples were preserved and stored until further analysis.

Additional surface seawater was collected for use in roller tank experiments and filtered using a 0.2 μm filter cartridge (Supor Acropak 200, Pall Corporation). Before their first use, fresh filter cartridges were rinsed in line with a minimum of 5 L of seawater, and before each sample collection, they were further rinsed with 0.5 L of seawater. The filtered seawater was stored in acid-washed High Density Polyethylene containers.

### Dissolved inorganic nutrients

Standard spectrophotometric method^87^ was used to measure dissolved inorganic silicate (DSi) and nitrate + nitrite (DIN) with the help of a UV-1900i Spectrophotometer (Shimadzu). Calibration was performed with standard reference solutions (KNO_3_ and Na_2_SiF_6_; Sigma Aldrich). Dissolved inorganic phosphate (DIP) was analyzed according to the UNESCO protocol^88^, using a K_2_HPO_4_ (Sigma Aldrich) standard.

### Phytoplankton cell density and enumeration

The phytoplankton community was quantified^89^ and identified^90^ at the beginning and end of the incubation experiment to determine how the clay treatment affected the community. An aliquot of 250 mL in triplicate from each treatment was collected in polycarbonate bottles and preserved with acid-Lugol’s iodine solution (1 mL; 1%) and kept undisturbed for 48 h. Following this treatment, phytoplankton cells concentrated at the bottom of the bottles were removed by carefully siphoning off 210 mL of supernatant solution. From the phytoplankton concentrate liquid, 1 mL was used to obtain phytoplankton cell counts using a Sedgewick cell-counting chamber (Hydrobias Kiel, Germany) under an inverted microscope (Zeiss Axiovert A1).

### Phytoplankton carbon biomass estimation based on cell volume

Carbon biomass from the most dominant phytoplankton species was determined by converting their cell volumes to carbon using previously established empirical relationships. Several studies^91–93^ have used this method as a well-established approach for estimating carbon biomass in phytoplankton. This assessment provides a lower bound on the amount of carbon removed by clay mineral amendments. It was done instead of the direct POC measurements which were not possible due to logistical constraints of our experiments. Briefly, the cell volumes of the predominant phytoplankton species were determined according to their geometric shapes. The geometric shape-to-volume relationships for diatoms and dinoflagellates were established by Sun and Liu^93^ and for *Phaeocystis* by Roussseau et al.^30^. For each species, 8–10 cells were used to calculate the average cell volume. The cell volumes were then utilized to estimate the carbon content of the phytoplankton by employing the formula^30,31^ y = aV^b^, where y is the carbon content (pg C cell⁻¹), V represents the optically measured cell volume (µm³). For diatoms and dinoflagellates, the constants a and b used are from Menden-Deuer and Lessard^31^, and for *Phaeocystis* from Rousseau et al.^30^.

### 18S rRNA gene amplicon sequencing of flocs

18S rRNA gene amplicon sequencing analysis of the flocs to identify the phytoplankton in the organoclay flocs. DNA was extracted from the floc samples using the Qiagen DNeasy PowerSoil Pro kit (Qiagen, Hilden, Germany) and the sequencing was conducted by CD Genomics (New York City, USA). The nucleic acid samples were subjected to amplification by quantitative PCR (qPCR) and high throughput sequencing of Illumina PE250. Amplicons were performed on a paired-end Illumina MiSeq platform to generate 300 bp Paired-end (PE) raw reads and each PE read was assigned a unique barcode. PE reads were merged using FLASH (V1.2.11,http://ccb.jhu.edu/software/FLASH/) Subsequently, quality filtering was performed on the sequence according to Fastp quality control process. DADA2^94^ and Deblur plugins in Qiime2 were used for quality control and Amplicon Sequence Variants (ASV) were generated. Taxonomy is assigned using a pre-trained Naive Bayes classifier and plugin, trained on the Silva 138 − 99% ASVs, and the specific taxa abundance in each sample is obtained. Microsoft Excel was used to visualize the taxa abundance.

### Total chlorophyll *a* (TChl*a*) analysis

Size fractionated chlorophyll *a* was evaluated for each type of phytoplankton community to understand the impact of clay addition. Ambient and incubated seawater was filtered in triplicate onto 25 mm Whatman GF/F filters (Adventac, Japan) and stored in amber-colored glass bottles at -80 °C for analysis in the onshore laboratory. After reaching the laboratory, the phytoplankton containing GF/F filters were crushed, followed by 8 mL of 90% acetone addition, and kept for 24 h at -20 °C for pigment extraction. After 24 h, the extracts were centrifuged for 10 min at about 2500 rpm. The resulting supernatant was used for TChl*a* analysis using a UV-Vis spectrophotometer (UV-1900i, Shimadzu) with a 5-cm light path cuvette according to the methods described in Parson and Strickland^95^.

### Transparent exopolymer particle (TEP) concentration determination

Ambient and post-incubated TEP analysis was conducted on board *RV Endeavor* according to the spectrophotometric method^96^. Briefly, 50 mL samples were filtered onto 0.4 μm nuclepore filters, then stained with Alcian Blue (pH = 2.5) and rinsed with 6 mL DI water. Filters were soaked in 6 mL of 80% sulfuric acid for 2 h. After 2 h, the solution was measured spectrophotometrically (UV-1900i, Shimadzu) in a 1 cm cuvette at 787 nm against an international standard (Acidic polysaccharide Gum Xanthan).

### Bacterial enumeration and identification

Bacterial enumeration and 16S rRNA gene amplicon sequencing were performed to analyze the microbial communities in clay treatments. Total bacterial counts were determined using epifluorescence microscopy and a Petroff-Hausser chamber^97^. Samples were fixed in 4% formaldehyde, concentrated by centrifugation, stained with DAPI, and visualized under 400x magnification using a Nikon Eclipse Ti microscope. DNA was extracted from seawater samples using the Qiagen DNeasy PowerWater DNA extraction kit, while the floc samples were extracted using the Qiagen DNeasy PowerSoil Pro kit. 16S rRNA gene amplicon sequencing was conducted at SeqCenter LLC (SeqCenter, Pittsburgh, Pennsylvania), targeting the V3/V4 regions. Sequencing was performed on a NextSeq platform, generating 2 × 310 bp paired-end reads. Data processing and analysis were carried out using R (version 4.3.0) with the DADA2 pipeline (version 1.28.0) for quality filtering and ASV generation. Taxonomic assignment was performed using the SILVA database (v138.1). Visualization of results was done using R packages; phyloseq (version 1.44.0) and ggplot (version 3.4.2).

### Copepod grazing and fecal pellet sinking rate experiment

The copepod grazing experiment commenced with selecting 40 adult *Calanus* copepods, maintained in clean, filtered seawater (0.2 μm) for four hours to ensure gut clearance. Concurrently, an algal stock solution of 30,000 cells mL^− 1^ was prepared in a 1 L bottle, with cell concentration verified using the Orflo Moxi Z (MXZ001) coulter counter (ORFLO Technologies, USA). Additionally, a mixture of *Rhodomonas salina* (CCMP 768) and 20 mg L^− 1^ clay was prepared in a 550 mL bottle. *R. salina* was specifically chosen as the algal diet to avoid adding extra weight to fecal pellets, thereby facilitating a clearer comparison between the sinking rates of algae-only pellets and clay-augmented pellets.

For the grazing experiment, three treatments, each comprising a 250 mL glass bottle, were prepared as follows: (a) Algae (control), (b) Algae + Clay (20 mgL^− 1^) + 15 Copepods (Treatment 1), and (c) Algae + (40 mgL^− 1^) + 15 Copepods (Treatment 2). These glass bottles were then placed on an algae wheel and incubated at 12 °C. Before introducing the copepods, initial algae concentrations in each jar were quantified using the Moxi coulter counter.

After 23 h, the glass bottles were removed from the incubator. Copepods and fecal pellets were separated using an 80 μm mesh filter and transferred into petri dishes containing seawater. Final algal concentrations in both control and treatment bottles were assessed using the Moxi coulter counter. The copepods were then carefully extracted from the Petri dishes using tweezers and returned to the *Calanus* culture. Fecal pellets from control and clay treatments were transferred into separate Petri dishes using a Pasteur pipette and counted. A subset of 100 pellets from both control and clay treatments were photographed for further analysis.

### Pellet sinking rate analysis

Pellet size was analyzed using ImageJ software^98^. For the settling velocity experiments, a total of 25 fecal pellets were randomly selected from each of the treatments and photographed for size analysis using ImageJ. Ensuring environmental consistency, the cuvette containing seawater for the sinking experiments was stabilized to prevent convection currents and to match the temperature and salinity conditions of the water surrounding the pellets. Pellets were individually introduced into the cuvette using a 1 mL glass pipette. Their descent was video-recorded using Nikon lens (Nikkor 105 mm f/1.8 AI-S), and the sinking velocities were subsequently calculated using ImageJ.

### Statistical analysis

One-way ANOVA with replication was performed in MS Office Excel (2019) to check the level of significance (95% confidence level; *p* < 0.05) between the treatments for phytoplankton and zooplankton data set. To determine any significant variations in the bacterial communities from the 16S rRNA gene amplicon sequencing, beta diversity was calculated by Bray-Curtis distances for each sample. Principal Coordinate analyses was performed using R package, phyloseq (version 1.44.0). Any significant differences were tested by non-parametric multivariate permutational analysis of variance (PERMANOVA) (vegan version 2.6.6).

## Electronic supplementary material

Below is the link to the electronic supplementary material.

Supplementary Material 1

## Data Availability

All data used in this study are provided in the manuscript in the main files as well as in the supplementary materials. All amplicon sequencing datasets are available in the Sequence Reads Archive (SRA) of the National Centre for Biotechnology Information (NCBI) under the bioproject numbers PRJNA1149738 (SRA numbers: SRR30296852 - SRR30296884) for 16S rRNA amplicon reads and PRJNA1149732 (SRA numbers: SRR30296890 - SRR30296893) for 18s rRNA amplicon reads.
