# Supplementary material for: Organoclay flocculation as a pathway to export carbon from the sea surface
Source: Sci Rep. 2024 Dec 10;14:28863. doi: 10.1038/s41598-024-79912-z (PMC11631952; doi:10.1038/s41598-024-79912-z)
Supplement: Supplementary file 1 — Supplementary Material 1 [file 41598_2024_79912_MOESM1_ESM.docx]

**Supporting Information for**

**Organoclay Flocculation as a Pathway to Export Carbon from the Sea Surface**

Diksha Sharma^1^, Vignesh Gokuladas Menon^1^, Manasi Desai ^1,2^, Danielle Niu^1,3^, Eleanor Bates^1,4^, Annie Kandel^1^, Erik R. Zinser^5^, David M. Fields^2^, George A. O’Toole^6^, Mukul Sharma^1*^

^1^ Department of Earth Sciences, Dartmouth College, New Hampshire, USA

^2^ Bigelow Laboratory of Ocean Sciences, Maine, USA

^3^ Current Add: First-Year Innovation & Research Experience (FIRE), Office of the Senior Vice President and Provost, University of Maryland, Maryland, USA.

^4^ Current Add: Department of Oceanography, University of Hawaii at Manoa, Honolulu, Hawaii, USA.

^5^ Department of Microbiology, University of Tennessee, Tennessee, USA

^6^ Geisel School of Medicine at Dartmouth, New Hampshire, USA

*Corresponding author, [mukul.sharma@dartmouth.edu](mailto:mukul.sharma@dartmouth.edu)

**This PDF file includes:**

Figures S1 to S4

Tables S1 to S5


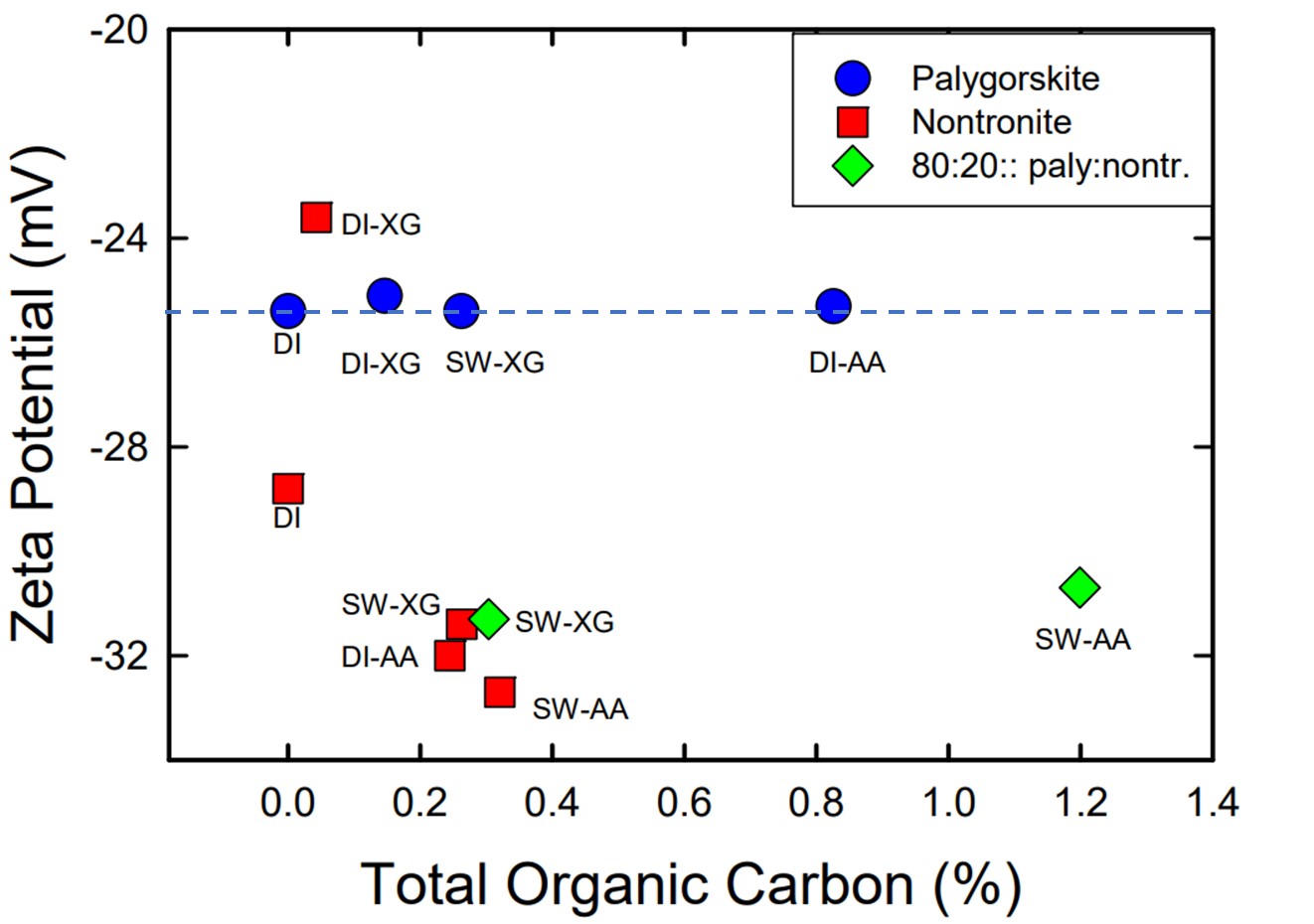


**Figure S1.** Change in ζ potential as clay minerals absorb acidic polysaccharides dissolved in synthetic seawater. In seawater, the clay mineral surfaces are negatively charged and attract positive ions from the solution creating a diffuse layer. The ζ potential is the voltage at the edge of the diffuse layer where it meets the surrounding liquid. The potential would change due to electrostatic interaction between clay and organic molecules. Palygorskite is a fibrous-clay with a point of zero charge (PZC) = 4.1, meaning that its surface is negatively charged at pH >4.1. Nontronite on the other hand has a PZC of 2.1. Note that an 80:20 mixture of palygorksite and nontronite seems to work synergistically to sorb more alginic acid from seawater than individual clay minerals. Here, SW = Seawater, DI = de-ionized water, XG = xanthan gum; AA = alginic acid.


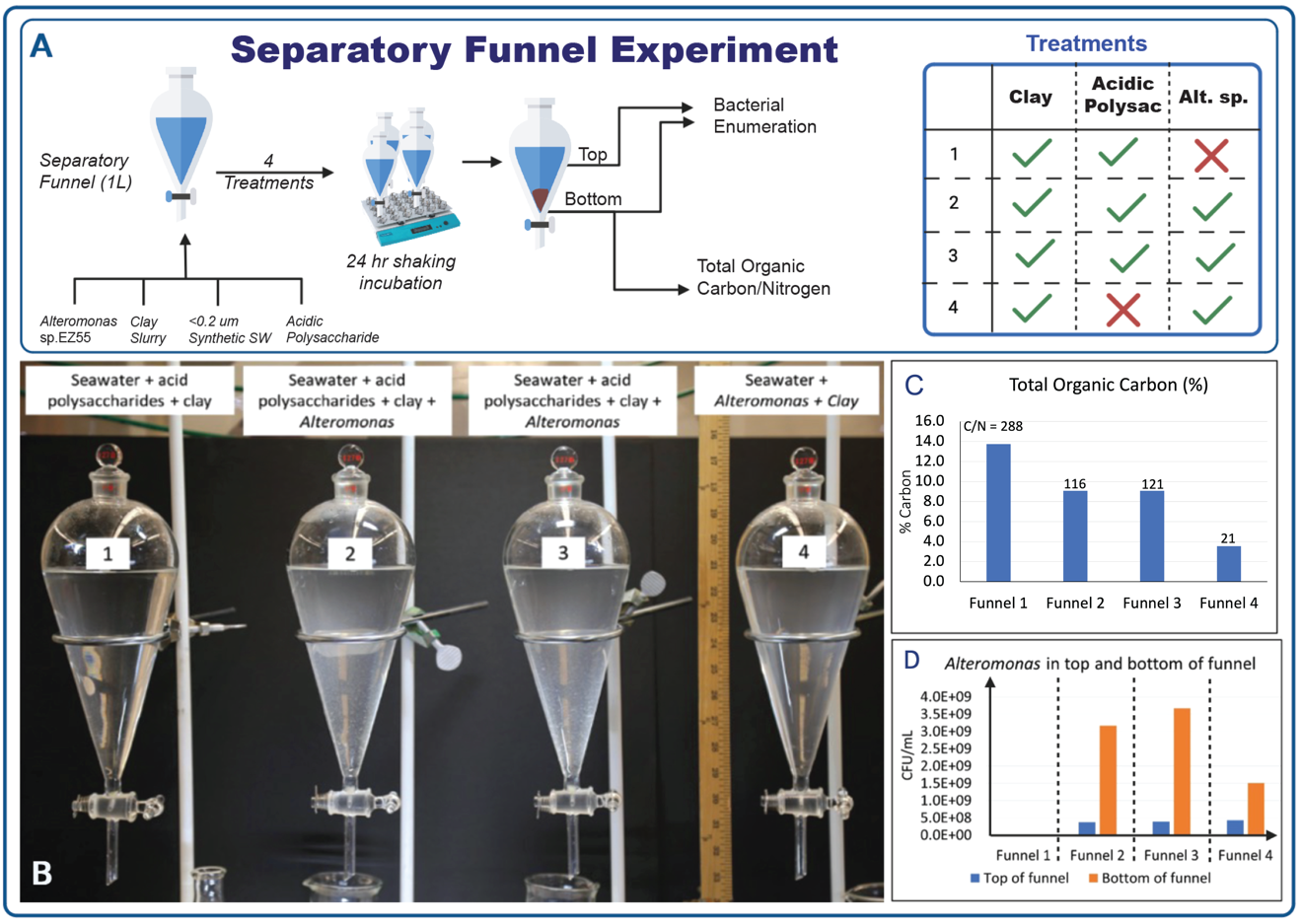


**Figure S2**. Separatory funnel experiments investigating the interaction of dissolved organic carbon, heterotrophic bacteria, and clay minerals. **(A)** The experimental protocol followed for the experiment, along with the 4 treatments investigated. **(B)** Each funnel contained 800 mL of synthetic sterile seawater (pH = 8.2; ionic strength = 0.72 M) in which a mixture of xanthan gum and alginic acid was dissolved (10 mg L^-1^). A natural clay mixture containing 80% palygorskite and 20% nontronite, with traces of apatite was sprayed on top of the funnel (20 mg L^-1^). Samples inoculated with *Alteromonas sp. EZ55* had an initial concentration of ~10^8^ CFU mL^-1^. Separatory funnels were shaken for 24 hr and then water and sediment samples were taken from the top and bottom of the funnels. The formation of flocs was visible in funnels (#3 and #4) with acidic polysaccharides and the bacterium when clay was sprayed **(C)** Percent total organic carbon and C/N ratios in the bottom sediment; the C/N ratio measured for freeze-dried *Alteromonas* is 5.3. After 24 hours organic carbon sorbed on clay surfaces is being metabolized by *Alteromonas*. **(D)** Colony Forming Units per mL (CFU mL^-1^) measured at the top and bottom of each funnel (1-4). These experiments showed that a) clay mineral mixture sorbs dissolved organic carbon (#1), b) Alteromonas preferentially sticks to clay surfaces (#4), and c) the bacterium concentration in organoclay flocs (orange) is over 10 times more than in seawater (blue) (#2 and #3).


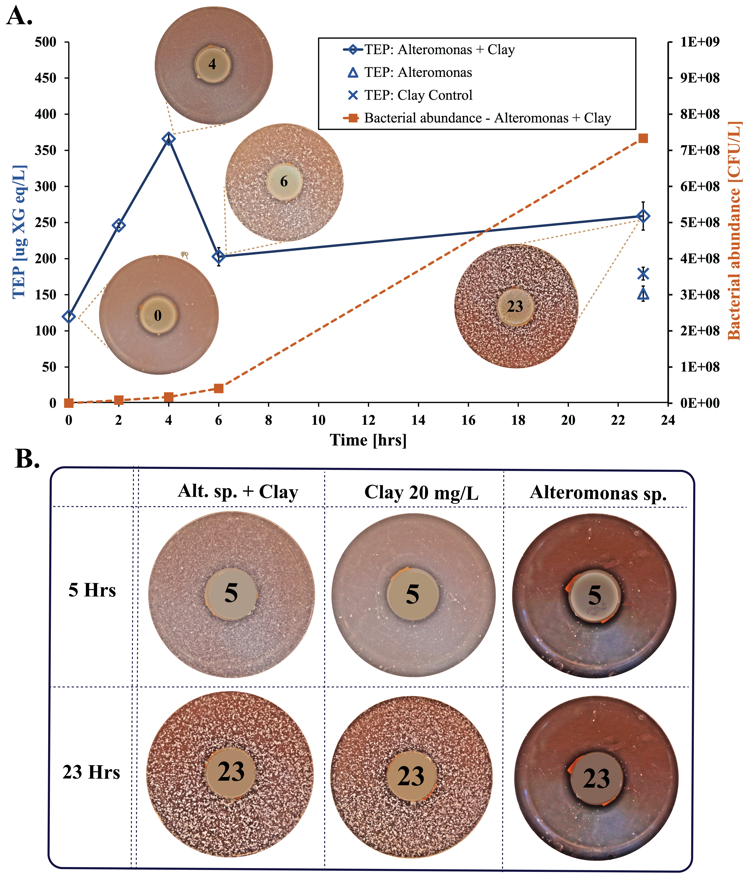


Figure S3. The interaction between *Alteromonas* sp. EZ55 and clay minerals in natural seawater was investigated in a roller tank experiment to understand the response of the bacterium to clay minerals in terms of TEP generation and flocculation. EZ55 was inoculated into 0.2 μm filtered seawater with 20 mg L^-1^ clay in a 1.15L roller tank and incubated at 3 rpm at room temperature for 23 hours. (A-B) Flocs were observed to begin forming within 5 hours in the bacteria + clay treatments when there was no significant clay flocculation observed in tank with just clay. There were no flocs observed in the treatments with only bacteria. *Alteromonas* sp. EZ55 treated with clay was observed to produce significantly higher TEP than just clay or *Alteromonas* only treatments after 23 hours of incubation.


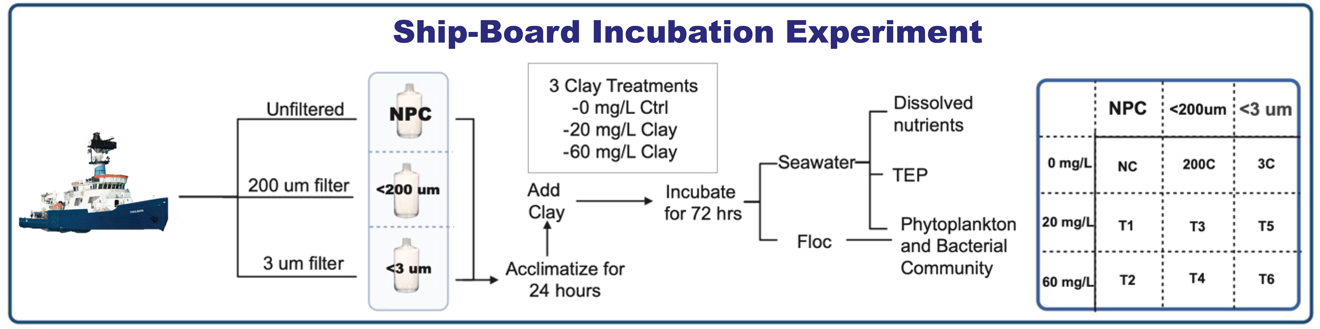


**Figure S4.** Schematic of incubation experiment conducted onboard *RV Endeavor* during the Spring 2023 phytoplankton bloom in the Gulf of Maine (GoM). Surface seawater was collected from Jordan basin, GoM using an acid-cleaned tow-fish system and split into three size fractions: Natural Phytoplankton Community (NPC), <200-PC community, and <3-PC community. The surface seawater was aliquoted in 1L polycarbonate bottles and plankton communities were allowed to acclimatize for 24 hours. Each of the bottles was then sprayed with a pre-determined amount of clay (0 mg L^-1^, 20 mg L^-1^, and 60 mg L^-1^) and incubated in a flow-through water bath on the deck under the natural diurnal cycle for 72 hours. Each sample was prepared in triplicate. Samples were collected post-incubation and analyzed immediately or preserved till analysis at home laboratory, as needed.

**Table S1.** Mineralogical composition of natural clay sample using quantitative XRD.

| **Minerals** | **Norm. Wt. %** |
| --- | --- |
| Quartz | 27.9 |
| Dolomite | 32.5 |
| Apatite | 6.7 |
| Nontronite | 6.8 |
| Palygorskite | 26.1 |

**Table S2.** Mean values of dissolved inorganic nutrients leaching from the clay addition in the seawater during a period of 72 hours (values ± SD; n = 3).

|  | **Silicate**  **(µmol L^-1^)** | **Phosphate**  **(µmol L^-1^)** | **Nitrate+Nitrite**  **(µmol L^-1^)** |
| --- | --- | --- | --- |
| **Natural Seawater** | 2.53±0.13 | 0.33±0.01 | 0.60±0.04 |
| **Clay 20 mg L^-1^** | 4.61±0.03 | 1.44±0.03 | 0.60±0.04 |
| **Clay 60 mg L^-1^** | 6.56±0.14 | 4.75±0.37 | 0.63±0.09 |

**Table S3.** Average values of dissolved inorganic nutrients, phytoplankton average cell density, total chlorophyll *a*, and TEP under experimental conditions (values ± SD; n = 3).

|  | **Silicate**  **(µmol L^-1^)** | **Phosphate**  **(µmol L^-1^)** | **Nitrate+ Nitrite**  **(µmol L^-1^)** | **Phytoplankton Cell no.**  **(×10^4^ L^-1^)** | **TChl*a***  **(mg m^-3^)** | **TEP**  **(µg XG eq. L^-1^)** | **Carbon content (mg C L^-1^) based on cell volume** |
| --- | --- | --- | --- | --- | --- | --- | --- |
|  | | | | | | | |
| **Initial** | 2.71±0.04 | 0.35±0.01 | 0.18±0.00 | 107 ± 1.13 | 6.13±0.28 | 263 ± 34 | 3.02 ± 0.18 |
|  | | | | | | | |
| **NPC Control** | 2.47±0.05 | 0.34±0.01 | 0.11±0.01 | 123.3 ± 2.4 | 7.34±0.41 | 472 ± 24 | 3.76 ± 0.13 |
| **T1 (20 mg L^-1^)** | 4.24±0.06 | 3.47±0.17 | 0.21±0.01 | 111.7 ± 1.7 | 6.69±0.55 | 4500 ± 76 | 3.19 ± 0.04 |
| **T2 (60 mg L^-1^)** | 6.22±0.43 | 8.05±0.34 | 0.30±0.03 | 89.1 ± 4.2 | 3.53±0.00 | 4712 ± 113 | 2.41 ± 0.07 |
|  | | | | | | | |
| **200µm Control** | 2.56±0.02 | 0.33±0.01 | 0.16±0.01 | 20.2 ± 0.9 | 3.19±0.06 | 408 ± 9 | 0.37 ± 0.03 |
| **T3 (20 mg L^-1^)** | 4.92±0.18 | 3.46±0.05 | 0.24±0.03 | 16.5 ± 1.3 | 1.81±0.07 | 3651 ± 180 | 0.21 ± 0.04 |
| **T4 (60 mg L^-1^)** | 5.88±0.43 | 6.05±0.22 | 0.23±0.00 | 14.8 ± 0.6 | 0.94±0.01 | 4420 ± 210 | 0.18 ± 0.03 |
|  | | | | | | | |
| **3µm Control** | 2.57±0.09 | 0.32±0.01 | 0.16±0.01 |  | 2.52±0.06 | 303 ± 5 |  |
| **T5 (20 mg L^-1^)** | 4.61±0.27 | 3.39±0.07 | 0.16±0.01 |  | 2.08±0.55 | 3449 ±173 |  |
| **T6 (60 mg L^-1^)** | 6.70±0.12 | 5.66±0.13 | 0.27±0.01 |  | 1.81±0.07 | 3798 ± 38 |  |

**Table S4.** Average cell abundances of individual phytoplankton species under experimental conditions (values ± SD; n = 3).

| **Phytoplankton classes** | **Phytoplankton species** | **Initial**  **(× 10^4^ L^-1^)** |  | **NPC**  **Control**  **(× 10^4^ L^-1^)** | **T1**  **20 mgL^-1^**  **(× 10^4^ L^-1^)** | **T2**  **60 mgL^-1^**  **(× 10^4^ L^-1^)** |  | **<200-PC**  **Control**  **(× 10^4^ L^-1^)** | **T3**  **20 mgL^-1^**  **(× 10^4^ L^-1^)** | **T4**  **60 mgL^-1^**  **(× 10^4^ L^-1^)** |
| --- | --- | --- | --- | --- | --- | --- | --- | --- | --- | --- |
|  | | |  |  | | |  |  | | |
| Dinoflagellates | *Tripos muelleri* | 39 ± 1.9 |  | 49.3 ± 1.4 | 42.1 ± 0.4 | 32.2 ± 0.8 |  | 4.8 ± 0.3 | 2.8 ± 0.5 | 2.2 ± 0.4 |
|  | *Tripos furca* | 5 ± 0.8 |  | 5.1 ± 0.6 | 3.4 ± 0.2 | 1.6 ± 0.2 |  | 0.3 ± 0.1 | 0.2 ± 0 | - |
|  | *Gymnodinium* sp. | 4.6 ± 0 |  | 6.6 ± 1.1 | 4.3 ± 0.5 | 2.5 ± 0.2 |  | 0.5 ± 0.1 | 0.5 ± 0.1 | 0.3 ± 0.1 |
|  | *Ostreopsis* sp. | 12.8 ± 3.1 |  | 10.8 ± 0.6 | 2.4 ± 1 | 1 ± 0.4 |  | 0.2 ± 0 | - | - |
|  | Unidentified dino | 3.9 ± 0.7 |  | 2.3 ± 0.1 | 1.8 ± 0.5 | 1.2 ± 0.5 |  | 0.6 ± 0.2 | 0.5 ± 0.4 | 0.3 ± 0.2 |
| **Total dinoflagellates** | | **65.3 ± 0.42** |  | **74.1 ± 1.6** | **54 ± 1.2** | **38.4 ± 1.7** |  | **6.3 ± 0.6** | **3.9 ± 0.8** | **2.8 ± 0.7** |
|  | | |  |  | | |  |  | | |
| Diatoms | *Chaetoceros* sp. | 5.7 ± 0.9 |  | 6.3 ± 0.4 | 16 ± 0.5 | 16.9 ± 0.6 |  | 3.6 ± 0.2 | 4.1 ± 0.2 | 4.4 ± 0.2 |
|  | *Thalassiosira* sp. | 7.1 ± 0.4 |  | 6.3 ± 0.3 | 9.1 ± 0.6 | 4.5 ± 0.7 |  | 1 ± 0.2 | 0.6 ± 0.2 | 0.3 ± 0.1 |
|  | *Pseudo-nitzschia* sp. | 2.9 ± 0.4 |  | 3.9 ± 0.5 | 1.6 ± 0.2 | 1.9 ± 0.4 |  | 0.5 ± 0.1 | 0.7 ± 0.2 | 0.4 ± 0.2 |
|  | *Rhizosolenia* sp. | 0.7 ± 0.4 |  | 0.6 ± 0.2 | 1.8 ± 0.9 | 1.1 ± 0.4 |  | 0.3 ± 0.1 | 0.2 ± 0 | 0.2 ± 0 |
|  | *Guinardia* sp. | 1.8 ± 0.5 |  | 6.3 ± 0.5 | 6.3 ± 1 | 10.1 ± 0.3 |  | 4.4 ± 0.5 | 4.5 ± 0.4 | 4.9 ± 0.1 |
|  | *Coscinodiscus* sp. | 1 ± 0 |  | 0.5 ± 0.1 | 0.6 ± 0.2 | 0.5 ± 0.1 |  | 0.3 ± 0.1 | 0.2 ± 0 | 0.13 ± 0.1 |
| **Total Diatoms** | | **19.2 ± 0.84** |  | **23.8 ± 1.2** | **35.4 ± 2.4** | **35.1 ± 1.7** |  | **10 ± 0.6** | **10.1 ± 0.3** | **10.2 ± 0.2** |
|  | | |  |  | | |  |  | | |
| Haptophytes | *Phaeocystis* sp. | 22 ± 0.3 |  | 25.2 ± 0.6 | 22.2 ± 0.4 | 15.5 ± 1.2 |  | 3.8 ± 0.3 | 2.5 ± 0.3 | 1.7 ± 0.1 |
|  | | |  |  | | |  |  | | |
| Cyanobacteria | *Trichodesmium* sp. | 0.5 ± 0.1 |  | - | - | - |  | - | - | - |
|  |  |  |  |  |  |  |  |  |  |  |
| **Total Phytoplankton** | | **107 ± 1.13** |  | **123.2 ± 2.4** | **111.7 ± 1.7** | **89 ± 4.2** |  | **20.2 ± 0.8** | **16.5 ± 1.3** | **14.8 ± 0.6** |

**Table S5.** Fecal production rate of copepod *Calanus finmarchicus* after clay ingestion.

| **Fecal Pellets Production Experiment (n = 3)** | **Pellets copepod^-1^ day^-1^** |
| --- | --- |
| Control | 25 ± 12 |
| 20 mg/L clay | 42 ± 16 |
| 40 mg/L clay | 43 ± 12 |
